# The *C*. *elegans* Connectome Consists of Homogenous Circuits with Defined Functional Roles

**DOI:** 10.1371/journal.pcbi.1005021

**Published:** 2016-09-08

**Authors:** Aharon Azulay, Eyal Itskovits, Alon Zaslaver

**Affiliations:** 1 Department of Genetics, The Silberman Life Science Institute, Edmond J. Safra Campus, Hebrew University, Jerusalem, Israel; 2 Ph.D. Program in Brain Sciences, Edmond and Lily Safra Center for Brain Sciences, Hebrew University, Jerusalem, Israel; ETH Zurich, SWITZERLAND

## Abstract

A major goal of systems neuroscience is to decipher the structure-function relationship in neural networks. Here we study network functionality in light of the common-neighbor-rule (CNR) in which a pair of neurons is more likely to be connected the more common neighbors it shares. Focusing on the fully-mapped neural network of *C*. *elegans* worms, we establish that the CNR is an emerging property in this connectome. Moreover, sets of common neighbors form homogenous structures that appear in defined layers of the network. Simulations of signal propagation reveal their potential functional roles: signal amplification and short-term memory at the sensory/inter-neuron layer, and synchronized activity at the motoneuron layer supporting coordinated movement. A coarse-grained view of the neural network based on homogenous connected sets alone reveals a simple modular network architecture that is intuitive to understand. These findings provide a novel framework for analyzing larger, more complex, connectomes once these become available.

## Author Summary

How can we understand the function of gigantic complex networks (e.g. the brain) based on connectivity data alone? We use the available full connectome of a nematode and apply new approaches to find that the neural network is made of structurally homogeneous neural circuits. These sets of neurons also appear in defined regions of the network where they may provide valuable functional roles such as signal integration and synchronization. Moreover, if we redraw the network considering these homogeneous sets alone, we reveal a simplified network layout that is intuitive to understand. As connectome data of higher brain systems are soon to be released our novel approaches can be immediately applied to studying these complex systems.

## Introduction

Systems neuroscience is reaching the stage where large connectomes are being mapped and ambitious collaborative projects are established to decipher the fundamental questions relating structure and function [1–4]. To name few are the current attempts to construct a large-scale computer simulation of the human brain [5–7], the development of various methods for obtaining whole-brain functional dynamics and connectivity maps [8,9], and others [10–12]. These massive efforts will yield gigantic networks composed of millions of inter-connected neurons. This poses a genuine challenge: how to analyze these perplexing connectomes such that functional principles can be extracted based on connectivity data alone.

Various approaches and theories had been developed to understand the structure–function relationship in neural networks [13–18]. Analyses of network properties, such as clustering coefficient and characteristic path length, revealed that neural networks are organized in a small-world topology, where the path length between any pair of nodes is relatively short [13,14]. In addition, neural networks, like many other biological networks, show a power law degree distribution in which the majority of the neurons are connected to relatively few partners, while a small fraction of the neurons are connected to exceptionally high number of other neurons [14]. A different approach to analyzing networks was to focus on the recurring building blocks embedded in networks [19–23]. These studies revealed that defined small building blocks, termed network motifs, are significantly overrepresented in biological networks, including the neural network of the round worm *C*. *elegans* [19,22–27]. Focusing on these small motifs allowed deciphering their potential functional roles in the network [20–23,25,28–30]. In addition, linear systems analyses have been used to predict functional sub-circuits purely based on network topology [26,31,32].

Recently, an intriguing observation was made in the rat somatosensory cortex. Using multiple electrode recordings, Perin and colleagues [33] showed that the wiring in layer 5 pyramidal cells obeys the common neighbor rule (CNR). According to this rule, the more common neighbors a pair of neurons shares, the more likely for this pair to be connected (A neuron X is considered a neighbor of neuron Y if X shares a chemical synapse or a gap junction with Y. A neuron is considered a common neighbor to a pair of neurons X,Y if it is connected directly to both X and Y, either via a chemical synapse or a gap junction). A similar principle was also observed in the rat visual cortex as simultaneous electrophysiological recordings from adjacent layer 2/3 pyramidal cells showed that connected pairs of neurons are more likely to share a common input [34,35]. Conversely, unconnected pairs share very little common inputs. Such an organization is thought to generate relatively independent subnetworks that are embedded within the larger-scale network architecture [33–36].

Here we aimed to elucidate whether the CNR is indeed an organizing principle in neural networks, and if so, to elucidate the functional roles that common neighbor sets of neurons may confer the network. To carry such analyses on the network-wide level, we focused on the sole fully-mapped neural network that is currently available–the *C*. *elegans* neural network. The connectome of *C*. *elegans* hermaphrodites consists of 302 neurons for which a complete wiring diagram is available, including number of synapses, spatial anatomical position, and the nature of these connections (chemical synapses or electrical gap junctions) [26,37–39]. Importantly, these data provide the unique opportunity for studying such structure-function relationships at the network-wide level, rather than focusing on specific cell types of selected brain areas only.

We show that the CNR is indeed an emerging property in the neural network of *C*. *elegans*. Moreover, sets of common neighbors form homogenous structures that appear in defined layers of the network confer valuable functional roles. Focusing on these sub-circuits reveals a simple functional architecture of the network that is intuitive to understand, and establishes a novel framework for studying functionality in, yet to come, bigger and more complex neural networks.

## Results

### The Common Neighbor Rule is an emerging feature in the neural network of *C*. *elegans*

**Fig 1:**
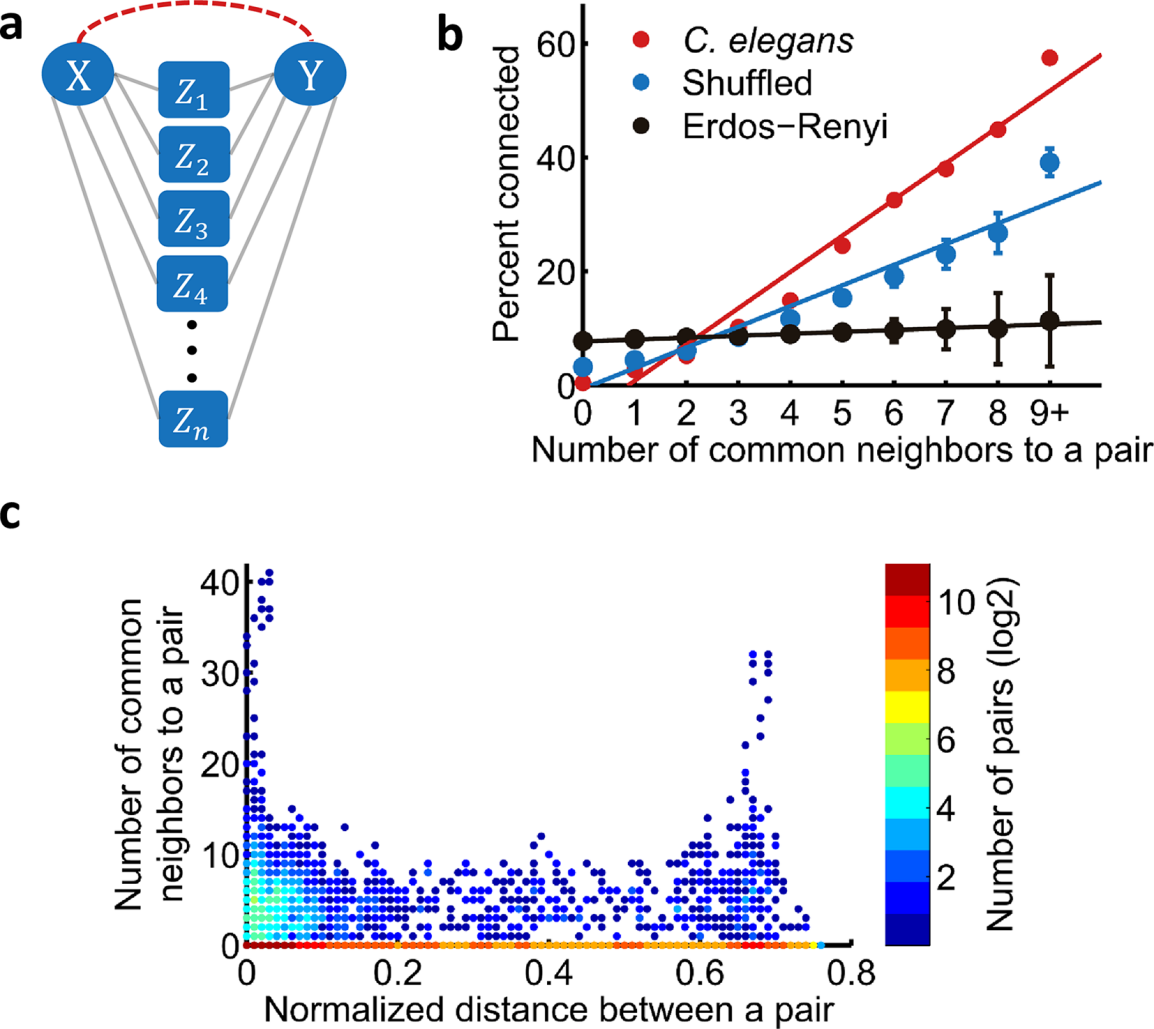
The common neighbor rule is an emerging property in the *C*. *elegans* neural network. (a) A set of common neighbors. According to the common neighbor rule, the more common neighbors (Z’s) a pair of nodes (X, Y) shares, the more likely for the pair to be connected (dashed, red). (b) The common neighbor rule is significantly more prominent in the *C*. *elegans* neural network (red) than in various random networks. Blue: random networks generated with the same degree distribution as the *C*. *elegans* neural network; Black: Erdős–Rényi random networks. Each random network has been fitted to a linear function, and the slopes were compared to the *C*. *elegans* slope (blue: R^2^ = 0.96, p<10^–10^; black: R^2^ = 0.94, p<10^–10;^ z-test). Error bars indicate standard deviation of 1,000 random networks. (c) The number of connected common neighbors to a pair is not biased by the physical distance between the pair of neurons (r = -0.13, p ≈ 1; One tailed student's t-test for Pearson correlation coefficient). Note the log scale of the color bar indicating that a large fraction of the connected pairs have zero common neighbors. The two peaks at short and long inter-somatic distances are due to the major head and tail hub neurons.

We begin by asking whether the CNR is found in the *C*. *elegans* neural network. To address this, we analyzed the available connectome of hermaphroditic *C*. *elegans* worms [26,37]. We find that the CNR is a striking feature in the *C*. *elegans* neural network where the fraction of connected pairs of neurons increase the more common neurons this pair of neurons shares (Fig 1A and 1B). In fact, this relationship grows linearly (R^2^ = 0.96) similarly to the relationship observed in the rat cortex [33].

The CNR observed in the *C*. *elegans* neural network could have arisen solely due to the degree distribution of the network. For example, the *C*. *elegans* neural network shows characteristics of a small-world network with a heavy tail degree distribution that follows a power law [14,26,38]. The CNR could have arisen merely because such networks contain hub neurons that connect many others. To test this, we generated random networks based on the known network topology but randomly shuffled the edges while preserving the in- and out- degree of each node constant [19] (S1 Text). While such random networks show CNR properties, the emergence of the rule in the genuine *C*. *elegans* neural network is significantly more prominent (p<10^–10^, z-test; Fig 1B). Finally, we analyzed whether random networks with no degree distribution constraints also obey the CNR. For this, we generated Erdős–Rényi random networks (S1 Text) and found that the CNR does not emerge in such networks (p<10^–10^, z-test; Fig 1B).

In neural networks, neurons are linked via physical connections in the form of chemical synapses or gap junctions. In particular, adjacent neurons are more likely to be connected than distant neurons since such a wiring strategy minimizes wiring costs [40–43]. Indeed, our analyses show a higher tendency to form connections between adjacent neurons (S1 Fig). Such distance-dependent connectivity may lead to local clusters in which neurons are more likely to be connected and share multiple common neighbors. To test if the CNR could have emerged solely due to this local clustering, we analyzed the number of common neighbors to a pair of neurons as a function of their inter-somatic distance. We find no correlation between these two parameters (Fig 1C, r = -0.13, p ≈ 1; One tailed student's t-test for Pearson correlation coefficient), thus excluding the possibility that physical proximity between neurons underlies the emergence of the CNR. In fact, we find that geometrically distant neurons can equally share multiple neighbors and that this feature depends on their degree (Fig 1C, notice the two peaks at short and long inter-somatic distances are due to the major head and tail hub neurons).

Together, these results demonstrate that the CNR is significantly overrepresented in the genuine neural network of *C*. *elegans*. Moreover, this rule cannot be explained by the networks’ degree distribution or by the spatial position of the neurons. This suggests that the CNR could evolve in the neural network probably as it confers functional roles.

### Sets of common neighbors are homogenous

**Fig 2:**
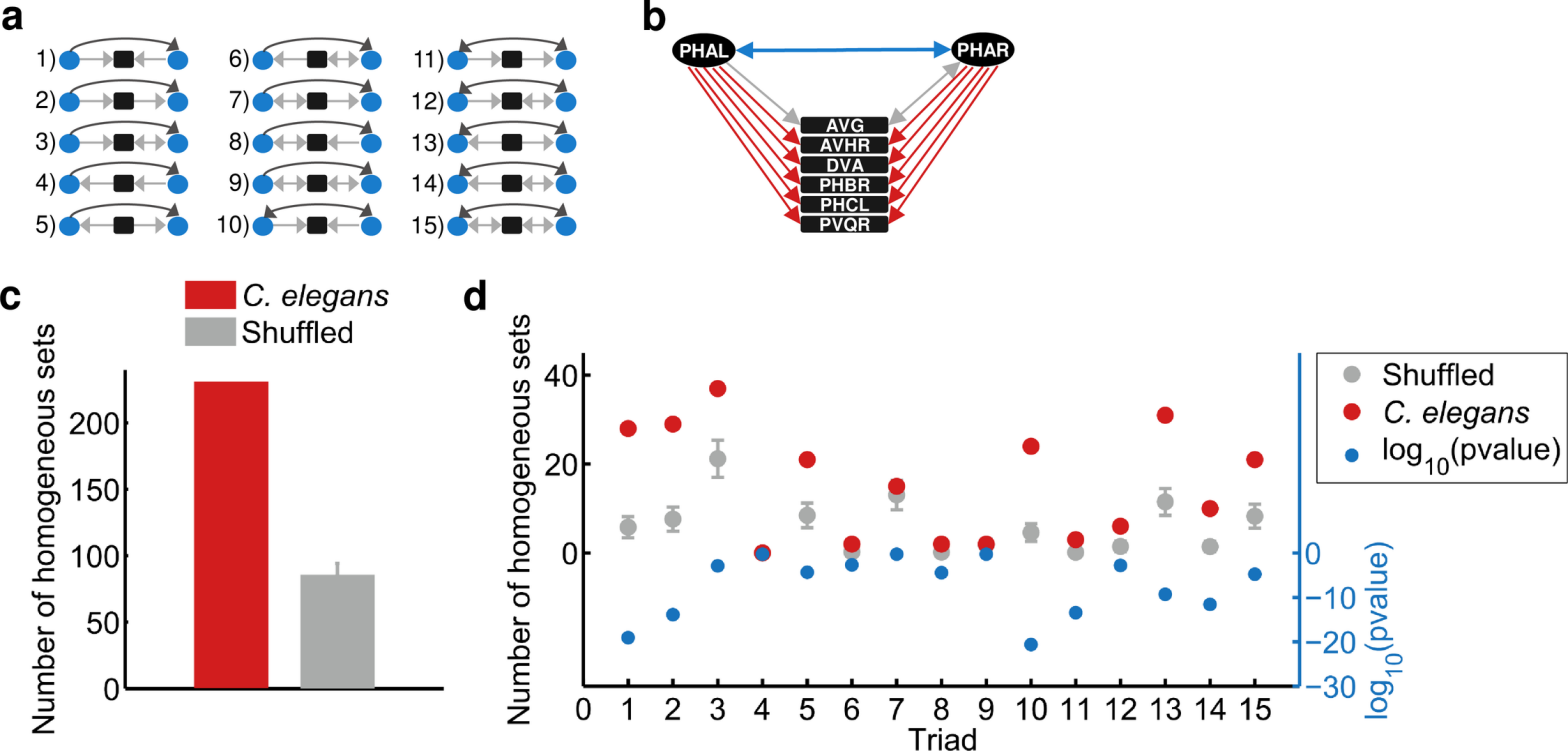
The *C*. *elegans* connectome is enriched with connected homogeneous common neighbor sets. (a) All possible connected triads when preserving the identity of connected X and Y pair of neurons and their mutual neighbors (Z’s). In triads 1–9, X and Y are connected uni-directionally, and in triads 10–15 X and Y are connected bi-directionally. (b) An example of a homogenous set of common neighbors where PHAL and PHAR are the X and Y neurons connected bi-directionally. Both PHAL and PHAR connect to multiple downstream neurons forming mainly type 10 triads. (c) The neural network of *C*. *elegans* is significantly enriched with homogenous sets when comparing to sets generated randomly by shuffling the existing sets (Hyper-geometric test p-value threshold is set to 0.05; p<10^–50^ for connected sets with five or more common neighbors). (d) Results of the same analysis shown in panel (c), but here the connected sets are grouped according to their specific type (according to panel (a)). The positive (left) y-axis represents the number of homogeneous sets in shuffled (grey) and real (red) sets. The negative (right, blue) y-axis depicts the significance of the difference between the shuffled and the real sets (z-test; after Bonferroni correction).

We next studied the structure of individual sets of common neighbors, where a set is a pair of neurons, X and Y, together with their common neighbors Z_1_, Z_2_,…Z_n_. A set can be connected or unconnected depending on the existence of a synapse between X and Y. Each set can be decomposed into *n* triads, such that each triad contains X, Y, and a single Z (*n* being the number of Z's; Fig 2A; S2A Fig). Theoretically, each set of common neighbors can be made of a mixture of the different triad types (for example, any of the triads 1–15 shown in Fig 2A for connected sets), resulting in a heterogeneous set structure. In such heterogeneous sets, the prospect to assign the entire set with a concrete functional role becomes virtually impossible since each triad type potentially carries distinct functional tasks. Surprisingly, however, we observed that most of the sets are not heterogeneous as randomly expected (examples of typical sets are shown in Fig 2B and S2 Table). To understand the tendency of a pair to become connected the more common neighbors it shares (the CNR) we continued by focusing on connected sets only (data concerning unconnected sets is shown in S2 Fig). Importantly, we focused on sets containing at least five common neighbors to minimize false positive homogenous sets formed by chance due to the small number of common neighbors.

To provide a quantitative measure for sets’ homogeneity we performed a statistical hyper-geometric test that takes into account the relative abundance of the triad type in all sets (S1 Text). Moreover, to extract the most significant homogeneous sets of common neighbors we introduced a second criterion on top of that defined by the hyper-geometric test: only sets of which at least half of their triads are of the same type are considered homogeneous. When filtering using these two very strict criteria, we find 231 (out of the 1,150 in total, ~20%) significantly homogeneous connected sets (hyper-geometric test p-value threshold is set to 0.05; S1 Text). The significance of sets’ homogeneity is further underscored when performing the same statistical analyses on randomly shuffled sets (p<10^–50^ when comparing *C*. *elegans* homogeneity to the homogeneity calculated for randomly shuffled sets, Fig 2C, S1 Text). Strikingly, these homogeneous connected common neighbor sets make a significant portion of the neural network comprising ~70% of the total synapses of the network. Of note, the vast majority of the homogeneous sets are not due to the bilateral symmetry of the neural network (we consider bilateral symmetric neurons only pairs of neurons of the form XXXR, XXXL; S3 Fig).

We next asked whether these homogenous connected sets of common neighbors are predominantly made of specific triad types. We find that only specific triad types are significantly overrepresented in homogenous sets (Fig 2D). In particular, sets homogenous with triads #10, #1, #2, #11, #14 and #13 make the top list among all sets that appear in the network significantly more than randomly expected, (triad #10 being the most significant and the others follow in descending order; p<10^–10^ for all; z-test after Bonferroni correction).

Interestingly, sets #1, #2 and #5 are made of triads forming a feed-forward loop (FFL), a known network motif in the *C*. *elegans* neural network [19,22,23,25]. Moreover, a topological generalization of the FFL circuit shows that these FFLs are embedded in larger clusters of multi FFLs [24]. These generalized FFLs resemble the homogenous connected sets of common neighbors that we observe in the network. One of the generalized FFL overrepresented in the *C*. *elegans* neural network is the multi-input FFL which corresponds to the homogenous common neighbor set made up primarily of triad #5. Indeed, and in agreement with Kashtan et al [24], our analyses show that this homogenous set is significantly overrepresented in the network (p<0.0001; z-test; after Bonferroni correction; Fig 2D). In addition, we find that the other FFLs are significantly overrepresented: multi-output and multi-inter FFLs which correspond to sets homogenous with triads #1 and #2, respectively (p<10^–10^; z-test; after Bonferroni correction; Fig 2D).

FFLs as well as their generalized multi-FFL forms had been previously studied emphasizing their potential functional roles in information processing in biological networks [19,24,29,30,44–46]. We find new homogenous set structures that appear significantly more than randomly expected and which had not been hitherto described in the context of neural networks. Among those, sets of type #10 and #13 are the most enriched with homogeneous sets (both in terms of the total number of sets, and in the difference from the shuffled sets; Fig 2D). The interesting feature in these two sets is the bidirectional connection between X and Y neurons. In triad #10, the X and Y neurons synapse one another and both are presynaptic to their mutual Z neurons, a structure termed mutually regulating (X and Y mutually regulate the Z neurons). In contrast, in triad #13, the bi-directionally connected X and Y neurons are post-synaptic to their mutual Z neurons, a structure termed mutually regulated (X and Y are mutually regulated by the Z neurons; Fig 2A).

### Connected homogeneous sets of common neighbors are confined to specific layers of the neural network

**Fig 3:**
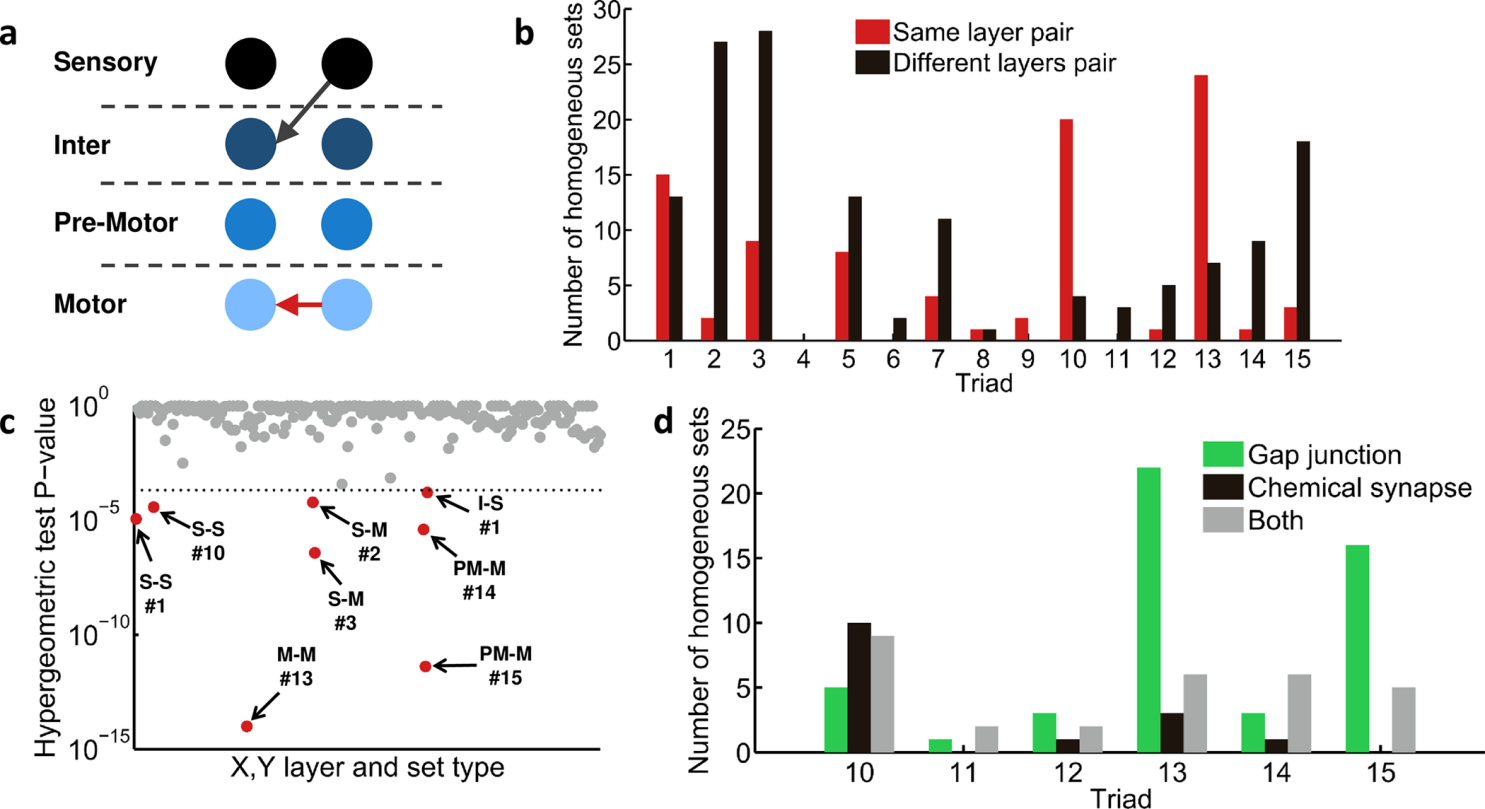
Connected sets of common neighbors are confined to specific layers of the neural network. (a) A common partitioning of the *C*. *elegans* neural network into layers. Black arrow indicates a neural connection between layers. A red arrow indicates a connection within the same layer. (b) For each connected homogeneous set type, the histogram shows how many times the pair of neurons {X,Y} are located either in the same layer or on different layers. (c) The significance (P-values of a hypergeometric test) of set type enrichment across the different layers. Note that only few (X-Y layers & set type) combinations crossed the significance threshold (p<0.005, hyper-geometric test, after Bonferroni correction). Layer notations: Sensory (S); Inter (I); Pre-Motor (PM); Motor (M). (d) Distribution of chemical synapses and gap junctions across all homogeneous sets of common neighbors in which X and Y have bidirectional connections (sets #10–15).

We proceeded by analyzing whether these sets appear in defined areas of the network. The rationale is that a circuit location can often hint of its potential functional role. For this, we defined four functional layers in the network and assigned each neuron to one of these layers based on its known function: sensory, inter, pre-motor, and motor neuron layers (for a complete list of neurons and their corresponding layers see S1 Table). Specifically, for each homogeneous connected common neighbor set, we analyzed whether the X and Y neurons are located on the same layer or on different layers of the network (Fig 3A and 3B). Interestingly, we found that X and Y neurons tend in general to reside on different layers, with the exception of the homogenous sets consisted of triads #10 and #13, the mutually regulating and mutually regulated sets, respectively (Fig 3B). In these two sets, the X and Y neurons are predominantly confined to the same layer. Moreover, in the mutually regulating sets (set type #10) both X and Y appear significantly more in the sensory layer than would be randomly expected (p = 0.009, hypergeometric test; after Bonferroni correction; Fig 3C and S4 Fig). In the mutually regulated sets (set type #13), X and Y appear almost exclusively in the motor neuron layer (p<10^–10^, hypergeometric test; after Bonferroni correction; Fig 3C and S4 Fig). Analysis of the type of the bidirectional synapse between X and Y reveals that in mutually regulating sets the bidirectional connection is enriched with chemical synapses, while in mutually regulated sets the bidirectional connection is made primarily of gap junctions (Fig 3D). Similarly, set #15, a fully bidirectional connected set (Fig 2A), that is significantly enriched with pre-motor and motor neurons (Fig 3C), is also made primarily of gap junctions (Fig 3D). To emphasize the enrichment of gap junctions in sets #13 and #15 we analyzed the network considering chemical synapses only. In such a network these sets are no longer overrepresented (p>0.05, S5 Fig). Overrepresented generalized FFLs, on the other hand, such as homogenous sets #1 and #2, show a different pattern of layer distribution where X and Y are distributed across all layers of the network (Fig 3B and 3C).

Taken together, we find homogenous sets of neurons, consisted of specific types of synapses, to appear in defined areas of the network. These observations may hint to possible functional roles of these sets in the neural network.

### Examples of homogenous sets and their potential functional roles in the neural network

To assign a functional role to such sets, we continue with the most significant homogeneous connected sets (Hypergeometric p-value threshold is set to 10^–5^, S2 Table). Here we provide two intriguing examples where linking the type of a homogenous set together with its network position and synaptic connections can disclose its potential functional roles in the network:

#### (1) Homogenous mutually regulating sets (sets of type #10)

In this type of sets, X and Y form a bidirectional connection (mostly involving chemical synapses) and both regulate multiple downstream neurons (Figs 2A, 2B and 3 and S6 Fig). Moreover, X and Y pair of neurons are enriched within the sensory neuron layer (Fig 3C). Of the most homogenous set of this type is the pair of sensory neurons PHAL-PHAR (S2 Table). A circuit with mutually synapsing sensory neurons, both regulating mutual downstream neurons, may have an important role in robust signal detection. In a case where the bidirectional feedback is positive, this circuit may amplify weak signals, integrate them over time, and thus serve as a short-term memory device. We simulated dynamics in such a circuit and indeed find that the existence of the bidirectional chemical synapse provides all the above functions (S7 Fig and S1 Text). Similarly, a circuit with a negative feedback may serve as a switch-like device ensuring that the downstream common neurons will respond to either X or Y, but not to both. The switch-like function can be particularly beneficial if X and Y carry opposite synaptic signs to Z (*e*.*g*., X activating and Y inhibiting the downstream Z neurons) [44].

#### (2) Homogenous mutually regulated sets (sets of type #13)

In this type of sets, X and Y form a bidirectional connection almost exclusively via gap junctions, and both are regulated by multiple mutual upstream neurons (Figs 2A, 3 and S6 Fig). Sets in which X and Y represent two inhibitory motor neurons are the most homogeneous among the type #13 mutually regulating sets (*e*.*g*., VD and DD motor neurons are electrically coupled sharing many mutual upstream neurons; S2 Table). This electrical coupling between motor neurons is found to be replicated along the body of the worm contributing to its sinusoidal motion [47,48]. As gap junctions have the characteristics of a low-pass filter preferentially transmitting sub-threshold potentials, they can contribute to synchronous activity of large neuronal ensembles [49,50]. Such synchronous activity is particularly relevant in the motoneuron layer where timely and balanced activity dynamics underlies the smooth coordinated undulation of the worm. Indeed, dynamics simulations of a simplified homogenous set of mutually regulated neurons demonstrates that a gap junction between X and Y neurons facilitates a more coordinated activity when compared to a circuitry in which the electrical coupling is absent (S8 Fig and S1 Text).

## Discussion

In this study we analyzed the connectome of the hermaphrodite *C*. *elegans* nematode and established that the CNR is an emerging property in this neural network. Strikingly, we find that specific sets of common neighbors are largely anatomically homogenous. Moreover, these sets are located in defined layers of the network indicating their potential functional roles in the neural network.

**Fig 4:**
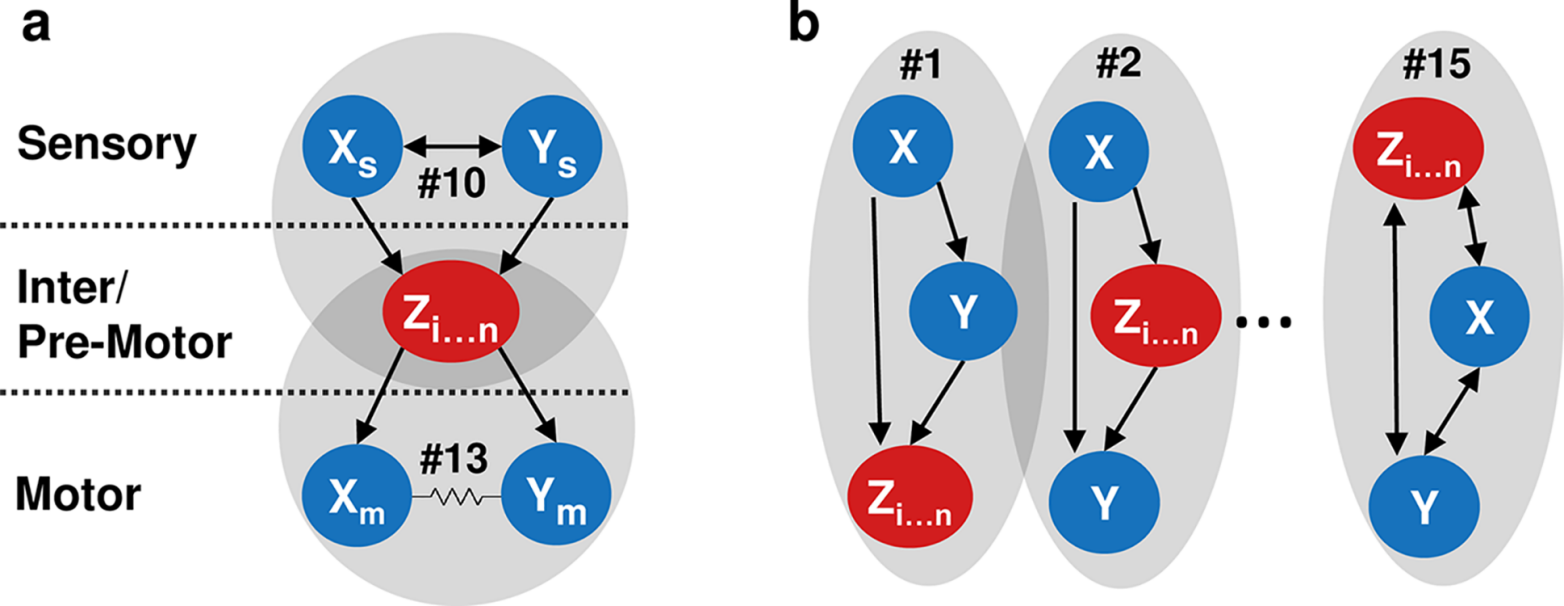
A coarse-grain reconstruction of the network based on homogenous sets of common neighbors reveals a simple network architecture that is intuitive to understand. (a) The mutually regulating and mutually regulated sets, triads #10 and #13, respectively, are found in defined layers: the mutually regulating sets (#10), enriched in the sensory layer, enable signal amplification and retention (short-term memory). The mutually regulated sets (#13), found almost exclusively at the motoneuron layer, support coordinated motoneuron activity that underlies the fine-orchestrated undulations. (b) Typical examples of additional significantly enriched homogeneous common neighbor sets. In these homogenous sets X and Y pair of neurons are mainly found on different layers of the network. Such sets can be particularly beneficial in mediating signal flow across layers. For example, sets #1 and #2 illustrate the typical Feed-forward Loop, a motif known for its potential functional roles in signal detection, filtering and more.

In fact, a coarse-grained view [51] of the neural network using the most abundant and the significantly overrepresented common neighbor sets reveals a simple network organization that is intuitive to understand (Fig 4). Specialized homogeneous sets appear in defined areas of the neural network, serving as functional building blocks that carry different processing tasks. For example, mutually regulating homogeneous sets support signal integration and amplification at the sensory/inter-neuron layers, while mutually regulated homogeneous sets synchronize multiple inputs from the common upstream neurons to support coordinated activity of the motor system (Fig 4A). Other sets, predominantly made of generalized forms of FFLs, are found throughout the network across different layers (Fig 4B). For example, set #1 is significantly overrepresented as a homogenous set in the network (S2 Table). In this set X and Y form a unidirectional chemical synapse, Y being mostly in the sensory layer, while X is either on the sensory- or inter- neuron layer (Figs 2A, 3 and S6 Fig). Of the most homogenous sets of this type is the pair of neurons AVHR-ADLR; ADLR being a sensory neuron and AVHR an interneuron (S1 and S2 Tables). The unidirectional chemical synapse from the AVHR interneuron to the upstream sensory neuron ADLR is an interesting feature that may provide a top-down feedback signal to modulate activity in a context dependent manner [52–56].

The coarse grain view suggests that the network can be partitioned and better understood based on these homogenous common neighbor sets. Such a partition contributes to a modular view of the network, and Indeed, biological networks are thought to evolve modular structures [16,33,36,38,45,57–61]. This modularity confers neural networks with robust learning capabilities [62], and rapid dynamic adjustments to constantly changing environments [24,45].

Our analyses revealed specific sets of common neighbors that are significantly overrepresented in the network: the mutually regulating and the mutually regulated sets. While these structures were previously studied in the context of developmental transcription programs [27,44,63], their significant emergence in neural networks was overlooked. This is possibly due to the different approach by which we analyzed the *C*. *elegans* neural network: (a) We considered the full wiring diagram currently available as opposed to previous analyses that considered neural connections with five synapses or more only; (b) The network we analyzed included gap junctions while previous analyses considered the network formed by chemical synapses only [24].

While providing unprecedented full connectome data, there are several limitations to the *C*. *elegans* wiring diagram. For example, it lacks important information regarding the directionality of gap junctions. In the absence of such data we considered all gap junctions as bidirectional, an obvious oversimplification of the genuine architecture. Similarly, the type and the nature of the chemical synapses are also largely unknown (for example, are the synapses excitatory or inhibitory; are they axo-dendritic, axo-axonic, dendro-dendritic, or dendro-axonic). In addition, the wiring diagram of the hermaphrodite *C*. *elegans* worm is reconstructed based on few animals only. In the lack of a greater number of reconstructed animals, one cannot be certain how wiring varies from animal to animal. Current efforts focus on reanalyzing the original EM data (WormWiring.org) [37], and to providing a map of the neurotransmitters expressed in each neuron [64]. These efforts will refine the connectome, so it will be interesting to apply our approaches once such data become available. Despite these obvious limitations, our comprehensive network approach focusing on the most significant homogenous sets, allows extracting meaningful circuits and assign them with potential functional roles.

Interestingly, social networks show similar connectivity patterns. In these networks, people interact and make connections preferentially with individuals that share similar backgrounds and interests (known as homophily [65]), and often add new friends if there is a common acquaintance (known as triadic closure [66]). Simulating social networks evolution with similar constrains yield networks characterized by the CNR [67]. In that respect, Facebook is a classic example for such a social network. We analyzed the available Facebook friendship connectivity and established that, indeed, the CNR is an emerging property in the Facebook social network: that is, the more common friends shared by two individuals, the more likely for these individuals to be friends as well (S9 Fig).

While the emergence of the CNR in social networks might be intuitive to understand, the benefit of such a design in neural networks is not trivially apparent. In the rat cortex the observed CNR is thought to be an organizing principle that clusters neurons into elementary building blocks of cortical computations and memory [33,36]. Our study provides several novel insights to this phenomenon: we established that the CNR is indeed an emerging organizing principle in the *C*. *elegans* neural network, and that sets of common neighbor neurons can be viewed as building blocks found in defined layers of the network exerting valuable functional roles (*e*.*g*., signal amplification, synchronization, and robust information processing). These novel findings may explain the emergence of the CNR in mammalian neural networks as well. For example, signal amplification and robust information processing are essential for efficient cortical computations. Thus, it will be fascinating to study these cortical building blocks in light of the observed CNR once such connectomes become available.

Importantly, we did not consider cell types a priori when analyzing the network to establish the CNR and the homogenous sets. Only subsequent analyses revealed the interesting principle of how information may flow through homogenous sets and across layers. In addition, homogenous sets are not found only between cells of different types, but many are between cells of the same type (For example set #10 appears more at the sensory neurons layer, while set #13 almost exclusively found at the motor neurons layer). Thus, applying these approaches to any new connectome may reveal hidden layers in what initially may seem as a homogenous layout made of the same types of neurons.

Finally, once connectome data of higher brain systems become available, the generality of our findings regarding network topology can be tested. Importantly, our approach can be used to extract the specific types of homogenous sets enriched in any connectome data, providing a novel platform to studying structure-function relationships in complex biological networks.

## Methods

A detailed description of all the methods used in this study can be found in the S1 Text file.

Briefly, statistical analyses and network randomizations were performed similarly to previous reports[19]. The connectome data (based on Varshney et al[26]) as well as the spatial position of the neurons along the anterior-posterior body axis[26,37] were obtained from: http://www.wormatlas.org/neuronalwiring.html.

For triad analyses, we generated an index of all possible types of connectivity between three neurons, preserving the identity of a neuron as either X, Y or Z (21 possible triads in total, Fig 2A, S2A Fig). To analyze homogeneous sets, we considered all connected pairs with five or more common neighbors (referred as sets of common neighbors), and performed a hypergeometric test (HGT) to retrieve the probability that a triad *j* appears *k(i*,*j)* times or more in the set *i* by chance; *k(i*,*j)* being the total number of triad of type j in set number i. A detailed description for triad analyses as well as the procedure to generate shuffled sets is found S1 Text.

We obtained the list of all neurons together with their description from: http://www.wormatlas.org/neurons/Individual%20Neurons/Neuronframeset.html. We assigned neurons to specific layers (sensory/inter/pre-motor/motor) based on their known function and anatomy (see S1 Table). To show that a particular set is enriched within a specific layer, we performed a HGT.

To simulate circuit dynamics we considered Michaelis-Menten type equations as *C*. *elegans* neurons typically show graded responses. The differential equations used in the simulations are detailed in S1 Text.

### Code availability

All code files generated in this study are available on github: http://AzulEye.github.io/HomogeneousSetsFinder.

The files include the code for generating all the figures in the manuscript as well as the random networks. In addition, we provide a generic pipeline that extracts homogeneous sets from any network using its adjacency matrix as an input. This is to be used with any future available connectomes or other biological networks such as transcriptional networks.

## Supporting Information

S1 Text A detailed description of all the methods used in this study. (DOCX)

S1 Fig Distance dependent connection probability. Closely positioned pairs of neurons are more likely to be connected (r = -0.54, p<10^–6^; One tailed student's t-test for Pearson correlation coefficient). Note the log scale of the color bar indicating that the vast majority of the connected pairs are relatively close in terms of inter-somatic distance. However, this tendency cannot explain the common neighbor rule as shown in Fig 1C.
(TIF)

S2 Fig The *C*. *elegans* connectome is enriched with unconnected homogeneous sets of common neighbors. (a) All possible unconnected triads when preserving the identity of connected X and Y pair of neurons (blue circles) and their mutual neighbors (black squares) (b) An example of an unconnected homogenous set of common neighbors. (**c**) The neural network of *C*. *elegans* is significantly enriched with homogenous sets when comparing to sets generated randomly by shuffling the existing sets (p<10^–50^, for unconnected sets with five or more common neighbors).
(TIF)

S3 Fig The formation of homogeneous common neighbor sets is not due to the bilateral symmetry of the neural network. In fact, among all X and Y pairs of neurons only a small fraction is actually bilateral (an example of a bilateral symmetric homogenous set is shown in Fig 2B). The ratio between symmetric vs asymmetric pairs among all homogeneous sets is 0.036, while the ratio between symmetric vs asymmetric pairs in all sets is 0.025. These similar ratios suggest that bilateral symmetry neurons are not enriched in homogenous sets, hence, formation of homogeneous common neighbor sets cannot be attributed solely to the bilateral symmetry of the neural network. Of note, we considered pairs of neurons as bilateral symmetric only if their names are in the form of XXXR, XXXL.
(TIF)

S4 Fig Number of homogenous sets across all set types depending on the layer in which X and Y reside. Shown is the complete data set that was used to generate Fig 3C. For each box in this matrix we performed a hypergeometric test (HGT) and the results of these tests are presented in Fig 3C.
(TIF)

S5 Fig The same analysis as performed in Fig 2D but when considering chemical synapses only and discarding electrical gap junctions. This analysis shows that 4/6 of the sets with mutual connections between X and Y are now not overrepresented (p>0.05). This is consistent with our analysis as shown in Fig 3D where the significance of homogeneous sets with mutual connections between X and Y is mostly due to gap junctions. This is particularly true for set #13 which is made primarily of gap junctions (that is between X and Y neurons). Conversely, set #10 is enriched with chemical synapses as evident from Fig 3D. These chemical connections are still overrepresented (p = 0.0495), although to a lesser extent than in the full network, since these sets contain gap junctions as well (Fig 3D). It is for this reason that we simulated set #13 with electrical junctions while set #10 was simulated with chemical synapses.
(TIF)

S6 Fig Layer position of the X,Y pair of neurons relative to their common neighbor Z neurons across the different set types. (TIF)

S7 Fig Mutually regulating sets can amplify brief weak signals and serve as short-term memory devices. (a) Top—a simplified diagram of a connected mutually regulating set. Bottom—Simulations of circuit dynamics (X, Y, Z_1_ and Z_2_) following a brief external stimulus S_x_. Grey Arrow represents a chemical synapse; Black arrow denotes a bidirectional chemical synapse. The positive feedback between X and Y amplifies a brief stimulus (S_x_) sensed by X only, facilitating the cross of a threshold (dotted horizontal line). In addition, the positive feedback supports a longer retention of the signal in the system acting as a short-term memory device. (b) Top—a simplified diagram of an unconnected mutually regulating set. Bottom—Simulations of circuit dynamics (X, Y, Z_1_ and Z_2_) following a brief external stimulus S_x_ using the exact same parameters used in (a). In the absence of the positive feedback, the downstream Z_1_ and Z_2_ neurons do not cross the same activation threshold, and their activity period is much shorter. In both, (a) and (b), we used the same parameters: *α* = *β* = *K* = 1 (see S1 Text).
(TIF)

S8 Fig Mutually regulated sets support coordinated activity in face of variability and noise. (a) Top—a simplified circuit of a connected mutually regulated set. X and Y share a gap junction (green line) and receive chemical synapses from Z_1_ and Z_2_ (grey arrows). Bottom—simulations of the circuit dynamics: In spite of unsynchronized activation of the upstream Z neurons, X and Y are activated more evenly when sharing a gap junction. (b) Top—a simplified circuit of an unconnected mutually regulated set. Bottom—simulations of the circuit dynamics. Note the much larger differences in the amplitude of X and Y neuron in the absence of a gap junction (when compared to (a)). To impose variability and possible noise we used: β(Z_1_ → X) = 2 × β(Z_1_ → Y); K(Z_1_ → X) = 10 × K(Z_1_ → Y). That is, Z_1_ activates X stronger than it activates Y by a factor of two, and with a ten-fold higher likelihood affinity. In addition, Z_1_ and Z_2_ are unsynchronized with respect to their activation time (see S1 Text).
(TIF)

S9 Fig The common neighbor rule is a property of social networks. The data is based on the Stanford Large Network Dataset Collection and can be downloaded from: http://snap.stanford.edu/data/egonets-Facebook.html. This network is an anonymized Facebook ego-network of a single user with all his/her friends together with all the connections among these friends. We compiled a network of 348 users that we analyzed in the same way as we analyzed the *C*. *elegans* neural network (Fig 1B). We found that the CNR is a significant property of social networks as well (linear fit, R^2^ = 0.95).
(TIF)

S1 Table A list of all neurons together with their assigned layer in the network (Sensory, Inter, Pre-Motor, Motor). Data compiled based on: http://www.wormatlas.org/neurons/Individual%20Neurons/Neuronframeset.html
(PDF)

S2 Table A list of the most significant homogeneous common neighbor sets (Hypergeometric test p-value threshold is set to 10^−5^). The first row is the neuron name, and the second is the triad type of a given Z neuron.
(PDF)
