# Supplementary material for: The C. elegans Connectome Consists of Homogenous Circuits with Defined Functional Roles
Source: PLoS Comput Biol. 2016 Sep 8;12(9):e1005021. doi: 10.1371/journal.pcbi.1005021 (PMC5015834; doi:10.1371/journal.pcbi.1005021)
Supplement: S1 Text — (DOCX) [file pcbi.1005021.s001.docx]

**Supplementary information**

**Generating random networks.** *Generating random networks with the same degree distribution as the C. elegans neural network;* We started with the known connectome[26,37], and shuffled the edges randomly as follows: We randomly chose two synapses of two different neurons (A and B), each connects to two other neurons (C and D). Only if A is connected to C but not to D, and B is connected to D but not to C, we switched their connections so that A will connect to D, and B will connect to C. We repeated this process 2000 times to generate a single random network. In total, we generated 1000 such random networks. This procedure ensures that the ensemble of random networks preserves the in- and out-degrees for each node. A similar algorithm was used by Milo et al [19].

*Generating Erdős–Rényi networks:* A graph is constructed by connecting nodes randomly. Each edge is included in the graph with a probability 0.035 and independently of all other edges. This probability was chosen because it produces similar number of edges as the genuine *C. elegans* neural network. In total, we generated 1000 such random networks.

**Connectivity and neuron physical location analyses**. We base our analyses on the data available in: <http://www.wormatlas.org/neuronalwiring.html>. These data contain information of both directionality and the number of connections via chemical synapses or electrical junctions in the nervous system. Moreover, the data contain one-dimensional spatial positions of the neurons (i.e., position of the soma centers) along the anterior-posterior body axis[26,37]. We included all connections among non-pharyngeal neurons except for CANL/R and VC6 which did not have obvious synapses. We also excluded from our analyses neuro-muscular junctions. Consequently, the model connectome included 279 neurons with 2194 chemical synapses and 1030 electrical junctions (electrical junctions considered as reciprocal connection and therefore counted twice). This wiring diagram of C. elegans was considered as a simple directed graph. The number of edges is taken to be one if there is one or more edges in the *C. elegans* wiring diagram, and zero otherwise. Asymmetric adjacency matrix was constructed to illustrate the synaptic connections among the 279 neurons. The matrix size was accordingly 279×279, and its sum represents the total number of synapses. The sum, 2992, is smaller than the number of gap junction and chemical synapses combined, because of the overlap between electrical junctions and chemical synapses.

**Common neighbor rule analyses.** We computed the number of common neighbors for all pairs of neurons using the following algorithm: First, we generated two matrices: (1) A matrix in which the rows represent the neurons, and the values in the columns 1-*S_i_* represent the *S_i_* neighbors that send synapse to neuron *i*. (2) The same as #1, but the values in the columns 1-*R_i_* represent the *R_i_* neighbors that receive synapse from neuron *i*. In order to find the number of common neighbors to a given pair we intersected two rows within each matrix, and rows between the two matrices. The algorithm iterates over all $\frac{279*278}{2}$ possible pairs and when a pair has Z common neighbors, it updates a counting matrix: the rows represent the number of common neighbors Z, and the columns are either connected or unconnected pairs. Percent of connected pairs for each number of common neighbors was calculated by dividing the first column of the counting matrix by the sum of the first and second column multiplied by 100.

**Triad analyses.** We created an index of all possible types of connectivity between three neurons, preserving the identity of a neuron as either X, Y or Z (21 possible triads in total, Fig 2a, S2a Fig).

**Homogeneous sets analysis.** We considered all connected pairs with five or more common neighbors (referred as sets of common neighbors), and performed a hypergeometric test to get p-values as follows:

N – Total number of triads in the network.

n(i) – the number of common neighbors in set number i.

K(j) – the total number of triads of type j in the network.

k(i,j) – the total number of triad of type j in set number i.

$$p(i,j)=\sum_{t=k\left( i,j \right)}^{n(i)} \frac{\binom{K\left( j \right)}{t}\binom{N-K\left( j \right)}{n(i)-t}}{\binom{N\left( j \right)}{n(i)}}$$

This produces a p-value that represents the probability that a triad *j* appears *k(i,j)* times or more in the set *i* by chance. Since the *C. elegans* has a small connectome, we observed some triads that appear only once in a particular set, and the sets considered enriched with these triads nonetheless. This is due to rare triads which can cause a set to pass the hypergeometric test just because of small number effects. Therefore, in order to ensure homogeneity and not only a mere enrichment, we introduced a second criterion: a set *i* is considered homogeneous with triad *j* if this triad makes more than 50% of the total triads in the set. This stringent condition is not necessary when considering large networks, and ensures that we only consider highly homogeneous sets of common neighbors in our analyses.

*Randomly shuffled sets.* We compared the homogenous sets in the genuine neural network to randomly shuffled sets to test for the significance of their overrepresentation which will also verify the HGT statistical analyses. For this, we shuffled triad types (*k*) between the different sets, preserving the number of common neighbors at each set (*n*) and the total number of triads of different types in the entire network (*K*). We made sure that shuffling would be only between sets with the same kind of synapse between the X,Y pair (bi- /uni- directional).

**Network layers analysis.** The data regarding functional layer of each neuron was retrieved from: <http://www.wormatlas.org/neurons/Individual%20Neurons/Neuronframeset.html>. We assigned the neurons to specific layers (sensory/inter/pre-motor/motor) based on their known function (Table S1). In order to show that a particular set is enriched within a specific layer, we used a HGT similarly to the homogeneous set analyses shown above, but with the following parameters:

N – Total number of connected sets with 5 or more synapses.

n(i) – the number of homogeneous connected sets enriched with triad number i.

K(j) – the number of connected sets in which the X and Y neurons are in layers shown in the y-label of figure S4 and denoted by j.

k(i,j) – the number of homogeneous connected sets enriched with triad number i, and their corresponding X and Y neurons are in layers shown in the y-label of figure S4 and denoted by j.

The resulting p-value represents the probability that a layer *j* (of the X and Y neurons as shown in figure S4) contains *k(i,j)* times or more sets of type i by chance.

**Simulations of circuit dynamics.** The equations used to simulate neural dynamics are based on sigmoid functions as *C. elegans* neurons typically show graded responses[67,68].

*Mutually regulating sets.* To simulate the mutually regulating sets (sets enriched with triad #10) we considered the circuit shown in the top panels of figure S6a,b. The time-derivative activities of the neurons are:

1. In the case of a positive feedback between X and Y (S7a Fig):

$$\frac{\mathrm{dX}}{\mathrm{dt}}=S_{x}+ \text{β}\text{∙Y/(Y+K) - }\text{α}\text{∙X}$$

$$\frac{\mathrm{dY}}{\mathrm{dt}}=\text{β}\text{∙X/(X+K) - }\text{α}\text{∙Y}$$

2. In the case X and Y are not connected (S7b Fig):

$$\frac{\mathrm{dX}}{\mathrm{dt}}=S_{x}\text{ - }\text{α}\text{∙X}$$

$$\frac{\mathrm{dY}}{\mathrm{dt}}= \text{- }\text{α}\text{∙Y}$$

In both cases the Z neurons are described by:

$\frac{dZ_{1}}{dt}=\text{β}_{XZ_{1}}\text{∙X/(X+K)}+ \text{β}_{YZ_{1}}\text{∙Y/(Y+K) - }\text{α}\text{∙}Z_{1}$

$\frac{dZ_{2}}{dt}=\text{β}\text{∙X/(X+K)}+ \text{β}\text{∙Y/(Y+K) - }\text{α}\text{∙}Z_{2}$

Where and $\text{β}_{XZ_{1}}\text{ = }\text{β}_{YZ_{1}}\text{ = 1.3∙β}$.

In these Michaelis-Menten type equations, representing graded neural activity, β denotes maximal neural activity; K is the activation level of the presynaptic neuron that leads to half of the maximal activity of the postsynaptic neuron; α is a self-relaxation parameter (activity decay) of the neuron. S_x_ denotes a brief stimulus sensed by neuron X. For example, in figure S6c,d: Sx=0.8 during t=4-6 time units and zero otherwise.

An interesting example for such a set is the two sensory neurons PHAL and PHAR which mutually synapse one another, and five out of their six triads are of type #10 (Fig 2b). What functional roles could this set confer? To address this question we simulated signal propagation in a simple version of this circuit with the above equations and parameters (S7a,b Figs, top). Here X and Y are mutually synapsing neurons and each of them also synapses onto two downstream neurons Z_1_ and Z_2_.

An external strong stimulus (S_x_) to neuron X would elicit a response that will propagate to the downstream neurons Z_1_ and Z_2_. However, if S_x_ is a brief weak stimulus then a transient activity of X without activating neuron Y may not be sufficient to elicit activity in Z_1_ and Z_2_ downstream neurons. A positive feedback between X and Y, however, can amplify the weak response elicited in neuron X. Moreover, we find that mutually regulating sets are enriched with sets for which both X and Y co-appear in the sensory/inter-neuron layer (S4 Fig), while Z neurons are either on the same or the downstream layer (S6 Fig). This circuit design can ensure efficient integration of the signal over time and a longer retention of the stimulus, thus serving as a short-term memory device (S7 Fig). The amplification and signal integration over time can then be propagated downstream to elicit stronger activation of the Z neurons (S7a Fig). If, however, X and Y were not mutually synapsing, this functionality of the set is lost since Y is not activated and as a result the Z neurons are only weakly activated for a much shorter time period, possibly not crossing their activation threshold (S7b Fig). Importantly, since we find this set to be significantly homogenous, signals will propagate uni-directionally from the sensory/inter-neuron layer (X and Y) to downstream layers (Z neurons).

Alternatively, in mutually regulating sets X and Y may form a negative feedback that may serve as a switch ensuring that the downstream Z neurons will respond to either X or Y but not to both[44]. The switch-like function can be particularly beneficial if X and Y carry opposite synaptic signs to Z (*e.g.,* X activating and Y inhibiting the downstream Z neurons). Such a design provides an efficient simultaneous switch in case Z neurons activity needs to alternate between two states. A chemical synapse which bears a sign (excitatory/inhibitory), rather than a gap junction, has the properties necessary to provide the possible amplification or the switch-like functions. This is consistent with our findings that the mutually synapsing X and Y neurons share chemical synapses in the vast majority of these sets (Fig 3d).

*Mutually regulated sets.* To simulate the mutually regulated sets (sets enriched with triad #13) we considered the circuit depicted in top panels of figure S7a,b. Here X and Y share a gap junction. These electrically-coupled neurons are considered to be equal in their volume since they consist of two motor neurons (X and Y) of the same type and similar size (e.g. DD1 and DD2). Ions flow between these neurons instantaneously. This is denoted in the equation by halving the activity level of each neuron since a current produced in one neuron immediately travels to the two other neurons. This charge flow is thus much faster than the activity of chemical synapses that obey a sigmoid function.

The time-derivative equations of neural activities are (S8a Fig):

$\frac{\mathrm{dX}}{\mathrm{dt}}=\text{β}_{Z_{1}X}\text{∙}Z_{1}\text{/(}Z_{1}\text{+K) + }\text{β}\text{∙}Z_{2}\text{/(}Z_{2}\text{+K) - }\text{α}\text{∙X + (}\frac{Y}{2}\text{ - }\frac{X}{2}\text{)}$

$\frac{\mathrm{dY}}{\mathrm{dt}}=\text{β}\text{∙}Z_{1}\text{/(}Z_{1}\text{+}\text{K}_{Z_{1}Y}\text{) + }\text{β}\text{∙}Z_{2}\text{/(}Z_{2}\text{+K) - }\text{α}\text{∙Y + (}\frac{X}{2}\text{ - }\frac{Y}{2}\text{)}$

In the case X and Y are not electrically coupled (S8b Fig):

$\frac{\mathrm{dX}}{\mathrm{dt}}=\text{β}_{Z_{1}X}\text{∙}Z_{1}\text{/(}Z_{1}\text{+K) + }\text{β}\text{∙}Z_{2}\text{/(}Z_{2}\text{+K) - }\text{α}\text{∙X }$

$\frac{\mathrm{dY}}{\mathrm{dt}}=\text{β}\text{∙}Z_{1}\text{/(}Z_{1}\text{+}\text{K}_{Z_{1}Y}\text{) + }\text{β}\text{∙}Z_{2}\text{/(}Z_{2}\text{+K) - }\text{α}\text{∙Y }$

In both cases the Z neurons are described by:

$\frac{dZ_{1}}{\mathrm{dt}}=\text{β}\text{∙S}_{Z_{1}}\text{/(}\text{S}_{Z_{1}}\text{+K)} \text{- }\text{α}\text{∙}Z_{1}$

$\frac{dZ_{1}}{\mathrm{dt}}=\text{β}\text{∙S}_{Z_{2}}\text{/(}\text{S}_{Z_{2}}\text{+K)} \text{- }\text{α}\text{∙}Z_{2}$

# Where, and $\text{β}_{Z_{1}X}\text{ = 2∙β}$; $\text{K}_{Z_{1}Y}\text{ = 10∙K}$

# $\text{S}_{Z_{1}}$ denotes a brief stimulation of Z_1_ and its level is 0.8 only during t=3-4 time units and zero otherwise.

# $\text{S}_{Z_{2}}$ denotes a brief stimulation of Z_2_ and its level is 0.8 only during t=5-6 time units and zero otherwise.

In mutually regulated sets, and in contrast to the mutually regulating sets, the X and Y neurons are almost exclusively found in the most downstream motoneuron layer (Fig 3c and S4 Fig). A typical example is shown in Table S2: two motor neurons, DD1 and DD2, are bi-directionally connected, and all their upstream common neighbors synapse onto them, all forming triads of type #13. Interestingly, in such sets, X and Y are connected primarily by electrical gap junctions rather than chemical synapses (Fig 3d).

Generally, gap junctions have the characteristics of a low-pass filter preferentially transmitting sub-threshold potentials. As such, gap junctions contribute to synchronous activity of large neuronal ensembles[49,50]. Synchronous activity is particularly relevant in the motoneuron layer where timely and even activity dynamics underlies the smooth coordinated undulation of the worm. Indeed, simulations of a simplified homogenous set of mutually regulated neurons demonstrates that a gap junction between X and Y neurons facilitates a more coordinated activity in face of possible stochastic activity in the upstream neurons (S8 Fig). For example, if X and Y are required to operate synchronously in face of an uneven input (in terms of both time and amplitude) from the upstream neurons Z1 and Z2, then the presence of the gap junction produces similar activation profiles with a greater overlap (S8a Fig). Absence of the gap junction yields deviating activations of X and Y that may lead to uneven contractions in the downstream adjacent muscles hence impairing the coordinated sinusoidal motion of the worm (S8b Fig). Thus, correlated activation is achieved due to the electrical coupling which evens out activities of the two individual neurons. This fast averaging can support the smooth motor motion of the worm in face of noisy upstream neural input.
