# Supplementary figures and images for: The C. elegans Connectome Consists of Homogenous Circuits with Defined Functional Roles
Source: PLoS Comput Biol. 2016 Sep 8;12(9):e1005021. doi: 10.1371/journal.pcbi.1005021 (PMC5015834; doi:10.1371/journal.pcbi.1005021)

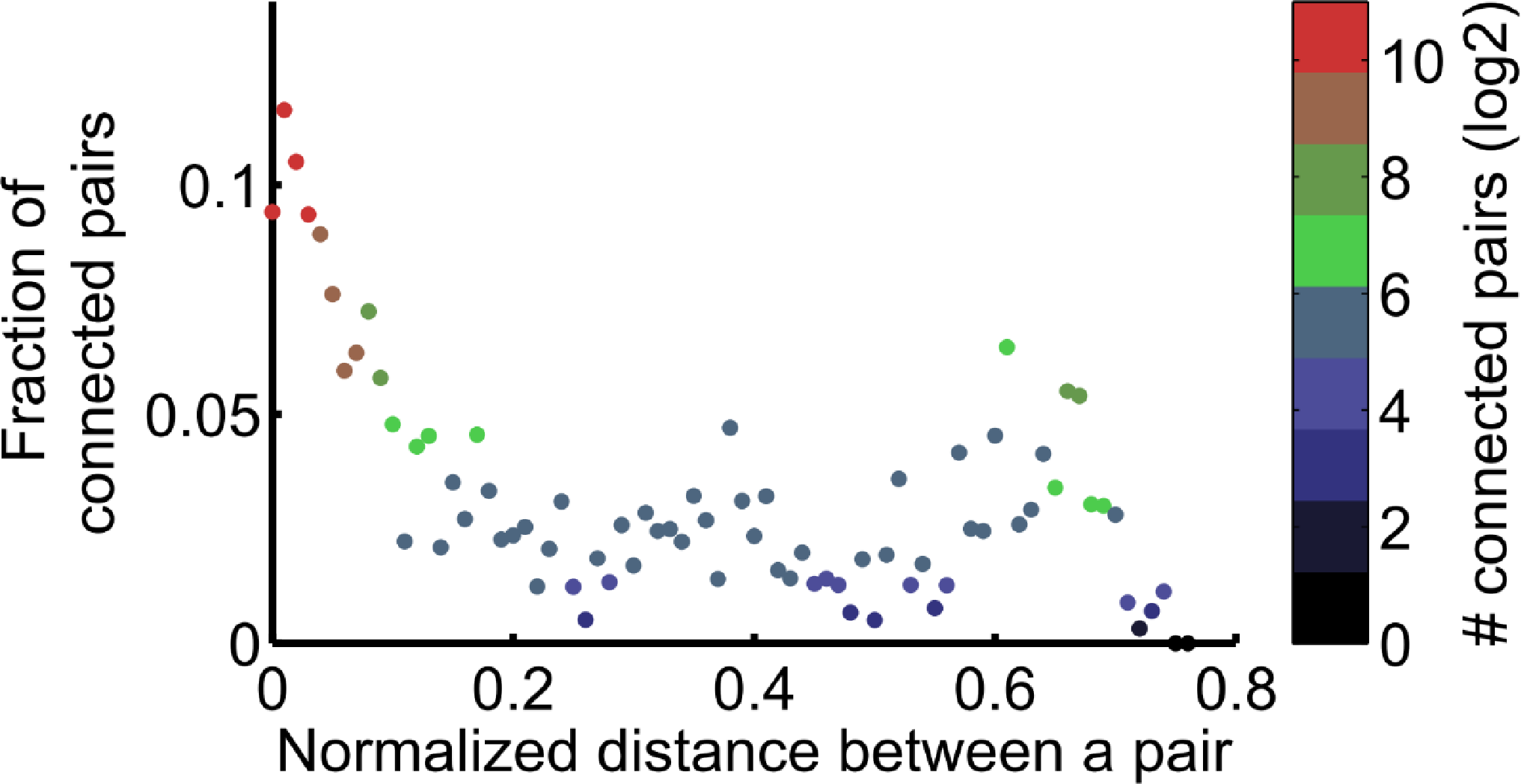

Supplement: S1 Fig — Closely positioned pairs of neurons are more likely to be connected (r = -0.54, p<10–6; One tailed student's t-test for Pearson correlation coefficient). Note the log scale of the color bar indicating that the vast majority of the connected pairs are relatively close in terms of inter-somatic distance. However, this tendency cannot explain the common neighbor rule as shown in Fig 1C. (TIF) [file pcbi.1005021.s002.tif]

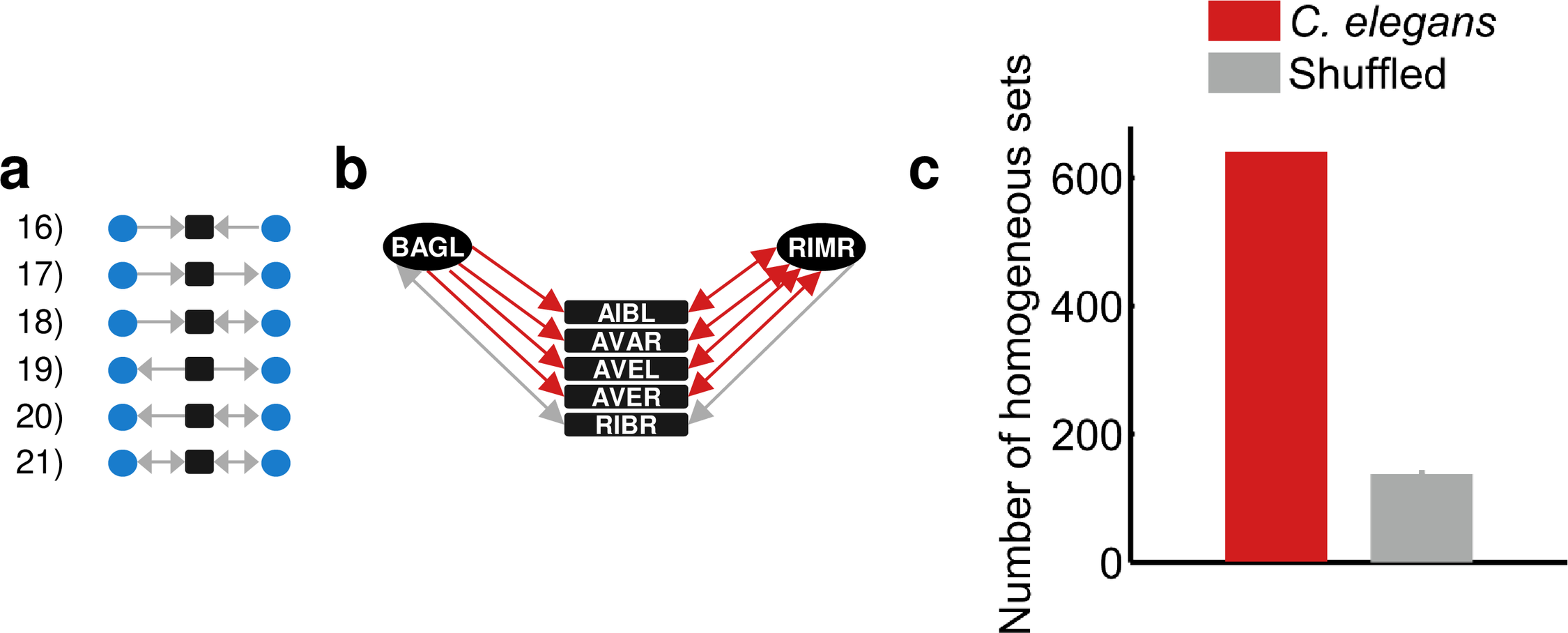

Supplement: S2 Fig — (a) All possible unconnected triads when preserving the identity of connected X and Y pair of neurons (blue circles) and their mutual neighbors (black squares) (b) An example of an unconnected homogenous set of common neighbors. (c) The neural network of C. elegans is significantly enriched with homogenous sets when comparing to sets generated randomly by shuffling the existing sets (p<10–50, for unconnected sets with five or more common neighbors). (TIF) [file pcbi.1005021.s003.tif]

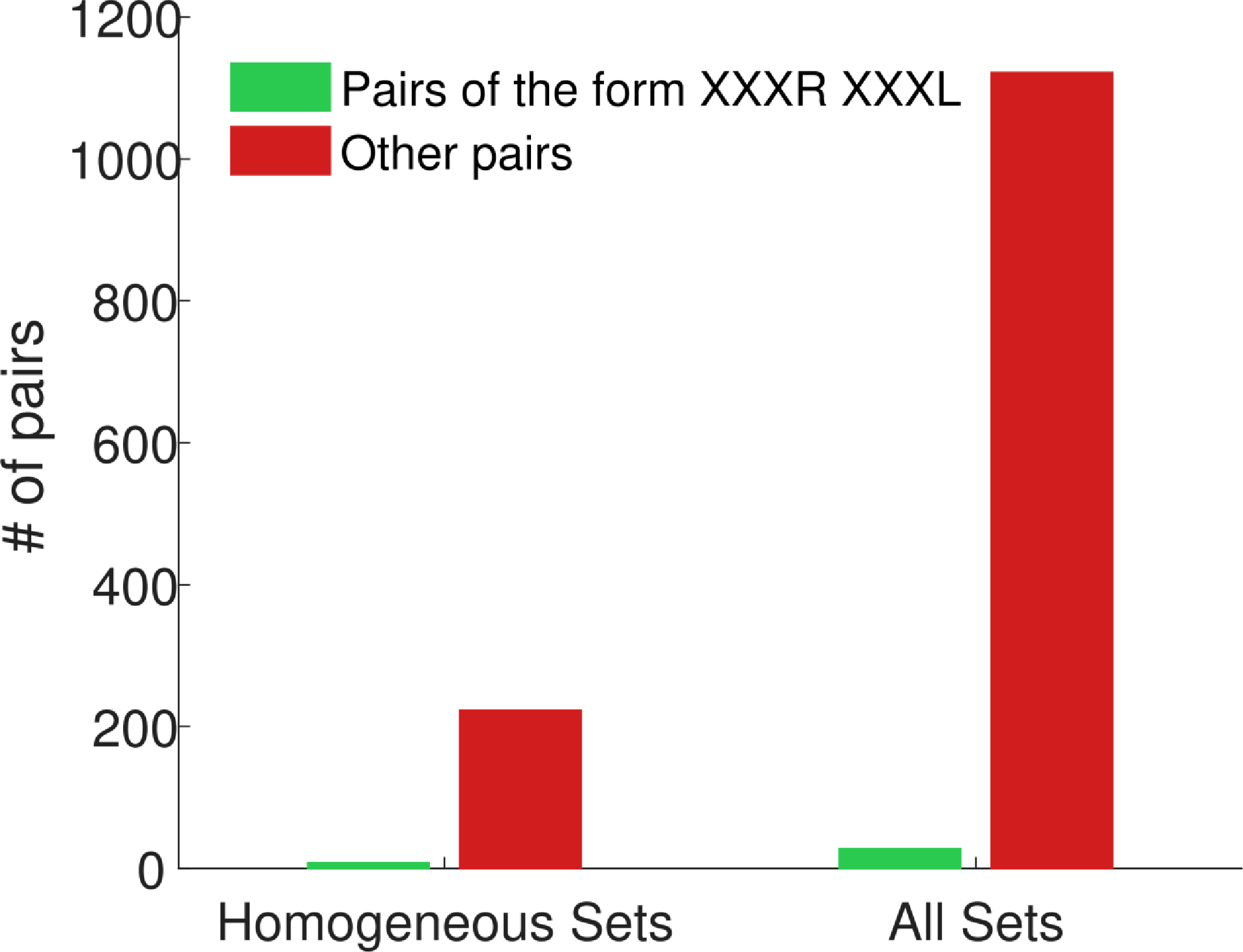

Supplement: S3 Fig — In fact, among all X and Y pairs of neurons only a small fraction is actually bilateral (an example of a bilateral symmetric homogenous set is shown in Fig 2B). The ratio between symmetric vs asymmetric pairs among all homogeneous sets is 0.036, while the ratio between symmetric vs asymmetric pairs in all sets is 0.025. These similar ratios suggest that bilateral symmetry neurons are not enriched in homogenous sets, hence, formation of homogeneous common neighbor sets cannot be attributed solely to the bilateral symmetry of the neural network. Of note, we considered pairs of neurons as bilateral symmetric only if their names are in the form of XXXR, XXXL. (TIF) [file pcbi.1005021.s004.tif]

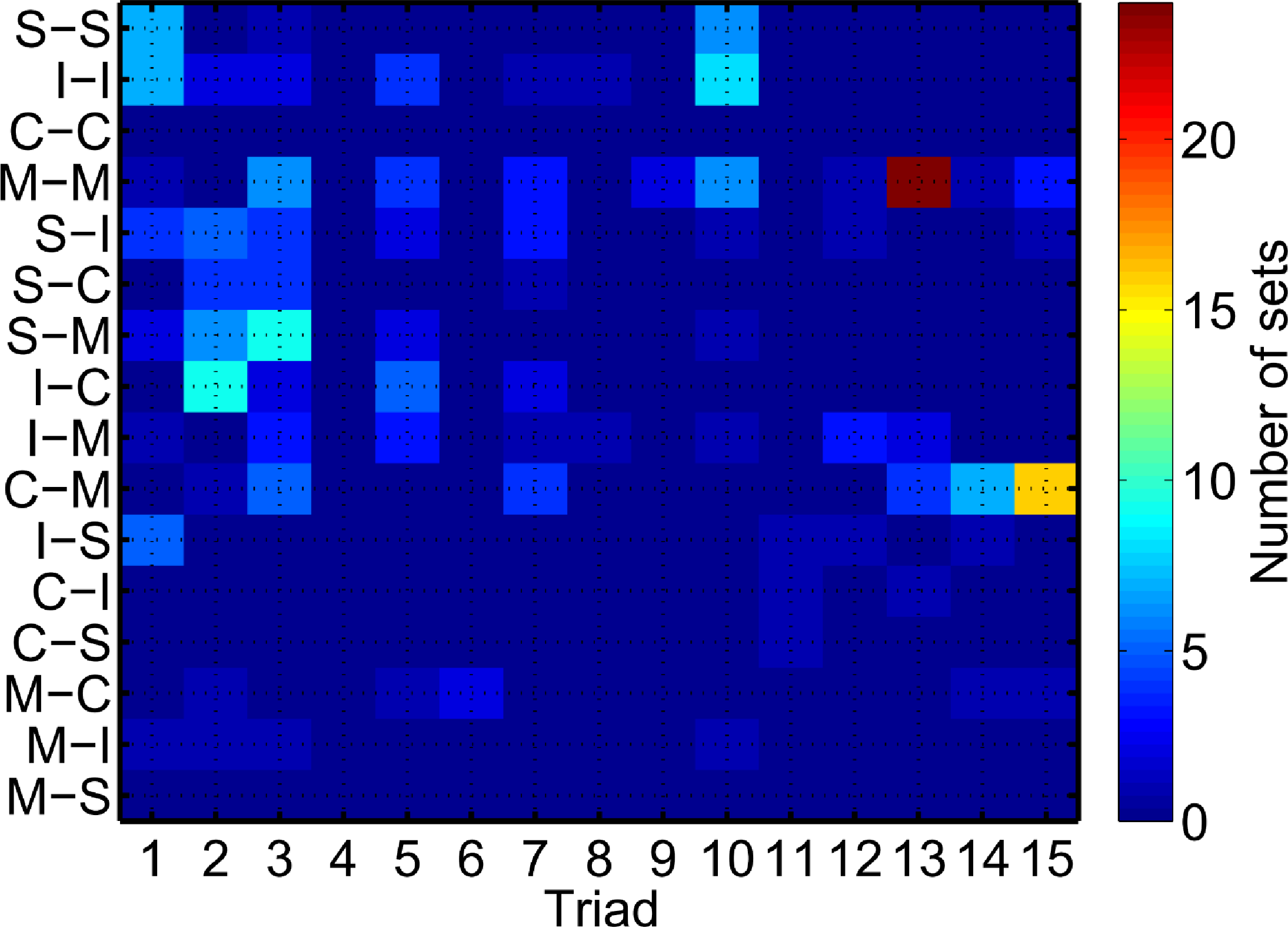

Supplement: S4 Fig — Shown is the complete data set that was used to generate Fig 3C. For each box in this matrix we performed a hypergeometric test (HGT) and the results of these tests are presented in Fig 3C. (TIF) [file pcbi.1005021.s005.tif]

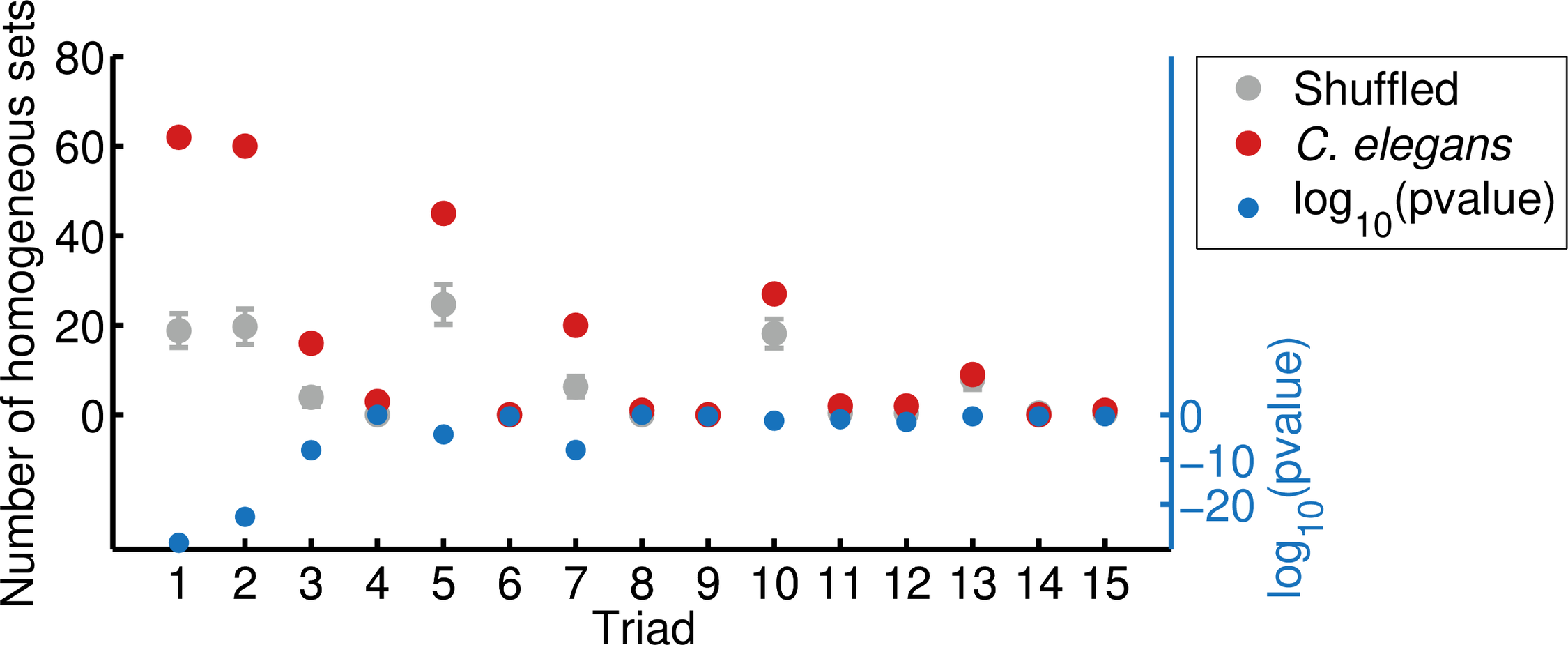

Supplement: S5 Fig — This analysis shows that 4/6 of the sets with mutual connections between X and Y are now not overrepresented (p>0.05). This is consistent with our analysis as shown in Fig 3D where the significance of homogeneous sets with mutual connections between X and Y is mostly due to gap junctions. This is particularly true for set #13 which is made primarily of gap junctions (that is between X and Y neurons). Conversely, set #10 is enriched with chemical synapses as evident from Fig 3D. These chemical connections are still overrepresented (p = 0.0495), although to a lesser extent than in the full network, since these sets contain gap junctions as well (Fig 3D). It is for this reason that we simulated set #13 with electrical junctions while set #10 was simulated with chemical synapses. (TIF) [file pcbi.1005021.s006.tif]

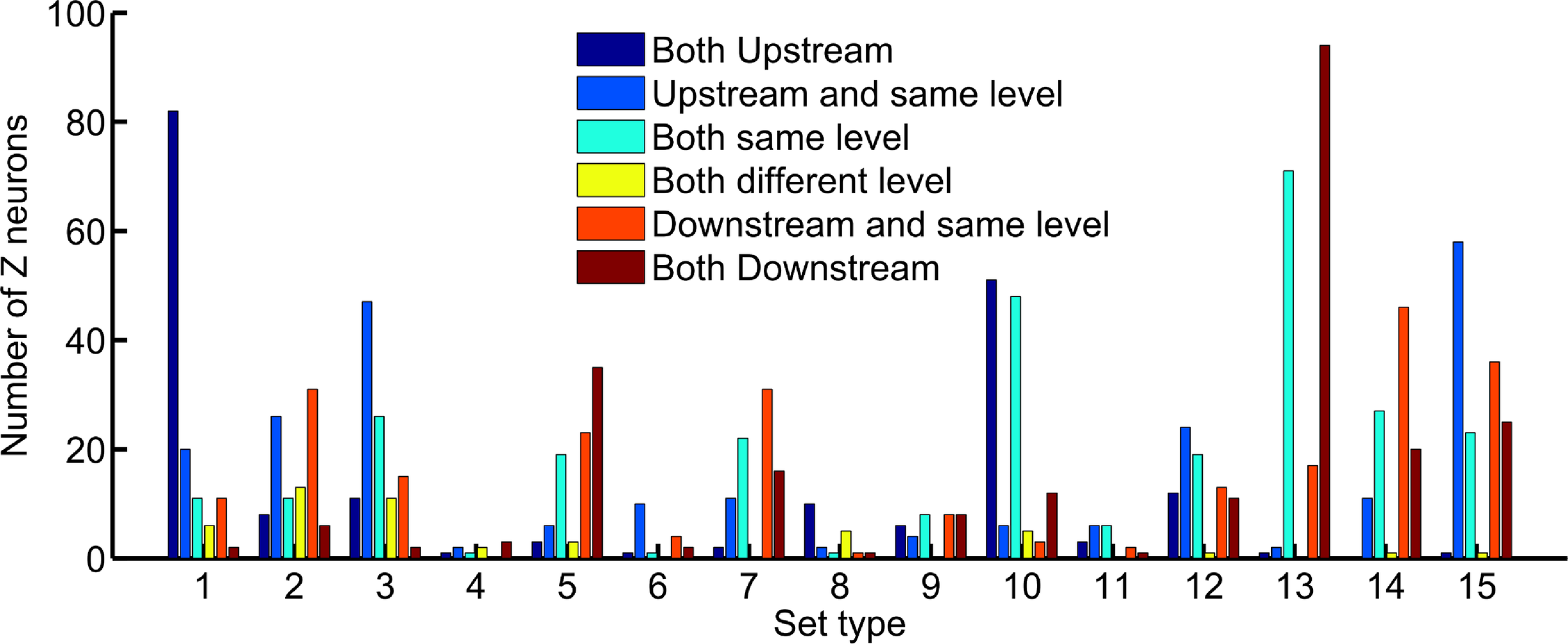

Supplement: S6 Fig — (TIF) [file pcbi.1005021.s007.tif]

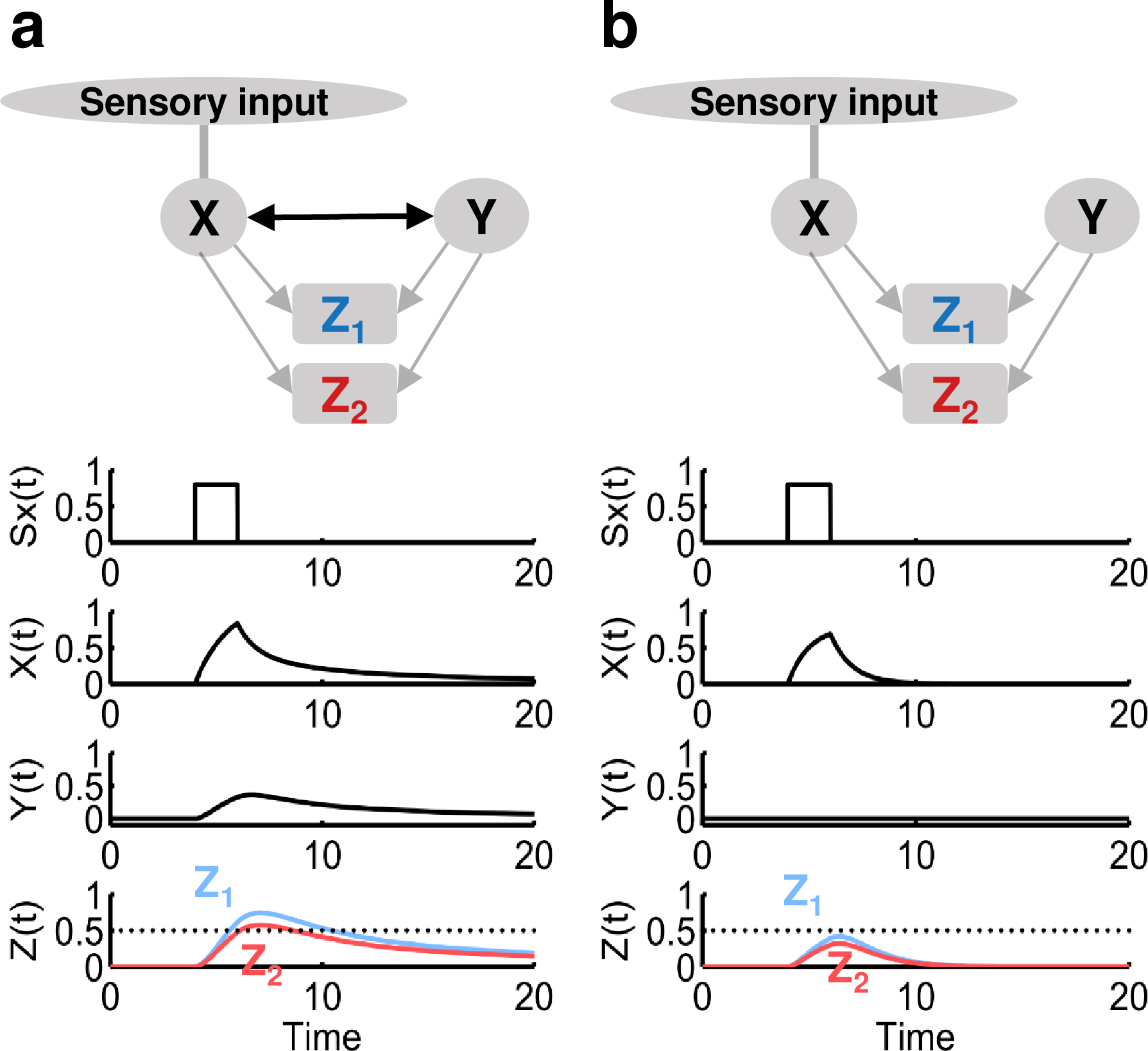

Supplement: S7 Fig — (a) Top—a simplified diagram of a connected mutually regulating set. Bottom—Simulations of circuit dynamics (X, Y, Z1 and Z2) following a brief external stimulus Sx. Grey Arrow represents a chemical synapse; Black arrow denotes a bidirectional chemical synapse. The positive feedback between X and Y amplifies a brief stimulus (Sx) sensed by X only, facilitating the cross of a threshold (dotted horizontal line). In addition, the positive feedback supports a longer retention of the signal in the system acting as a short-term memory device. (b) Top—a simplified diagram of an unconnected mutually regulating set. Bottom—Simulations of circuit dynamics (X, Y, Z1 and Z2) following a brief external stimulus Sx using the exact same parameters used in (a). In the absence of the positive feedback, the downstream Z1 and Z2 neurons do not cross the same activation threshold, and their activity period is much shorter. In both, (a) and (b), we used the same parameters: α = β = K = 1 (see S1 Text). (TIF) [file pcbi.1005021.s008.tif]

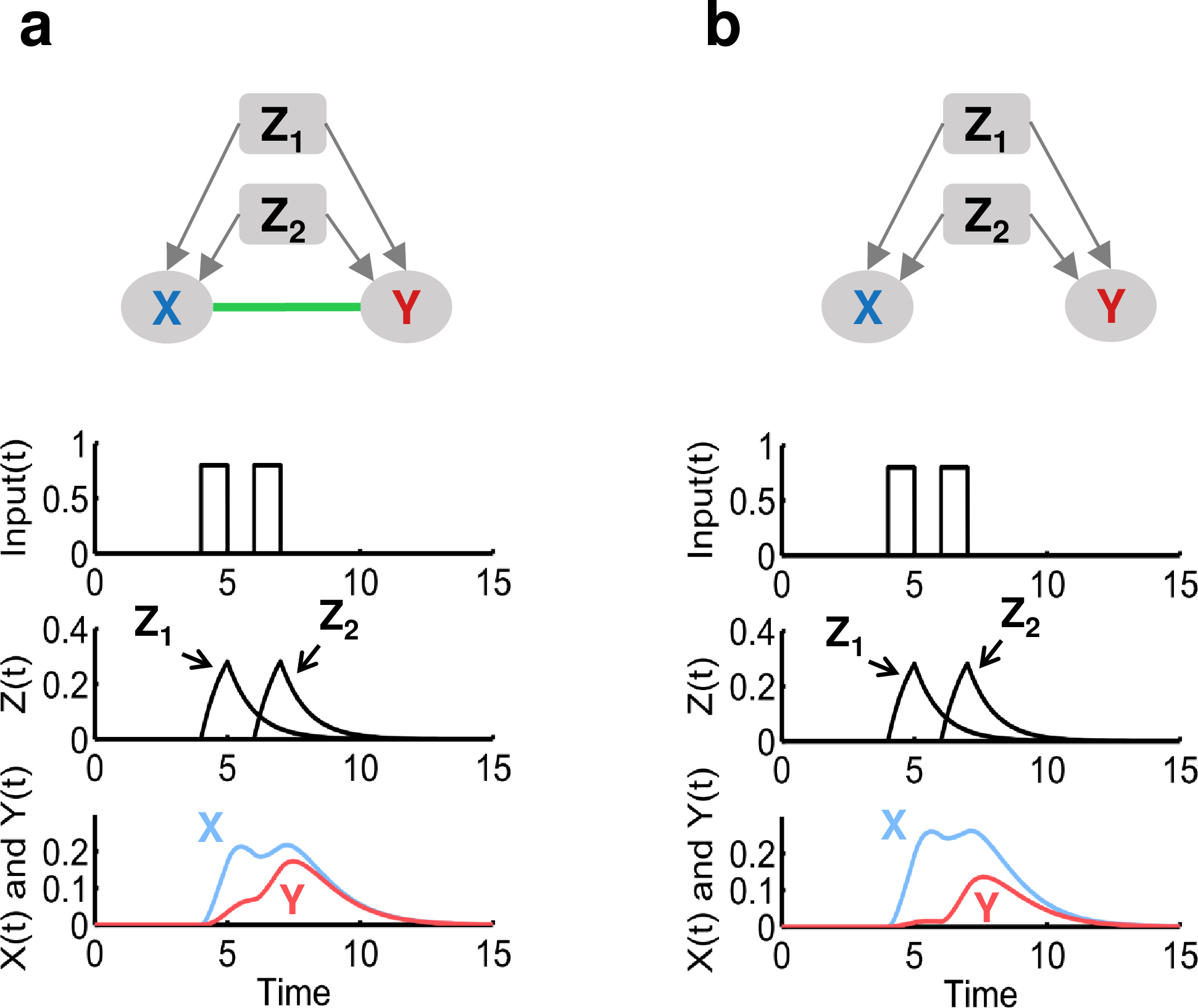

Supplement: S8 Fig — (a) Top—a simplified circuit of a connected mutually regulated set. X and Y share a gap junction (green line) and receive chemical synapses from Z1 and Z2 (grey arrows). Bottom—simulations of the circuit dynamics: In spite of unsynchronized activation of the upstream Z neurons, X and Y are activated more evenly when sharing a gap junction. (b) Top—a simplified circuit of an unconnected mutually regulated set. Bottom—simulations of the circuit dynamics. Note the much larger differences in the amplitude of X and Y neuron in the absence of a gap junction (when compared to (a)). To impose variability and possible noise we used: β(Z1 → X) = 2 × β(Z1 → Y); K(Z1 → X) = 10 × K(Z1 → Y). That is, Z1 activates X stronger than it activates Y by a factor of two, and with a ten-fold higher likelihood affinity. In addition, Z1 and Z2 are unsynchronized with respect to their activation time (see S1 Text). (TIF) [file pcbi.1005021.s009.tif]

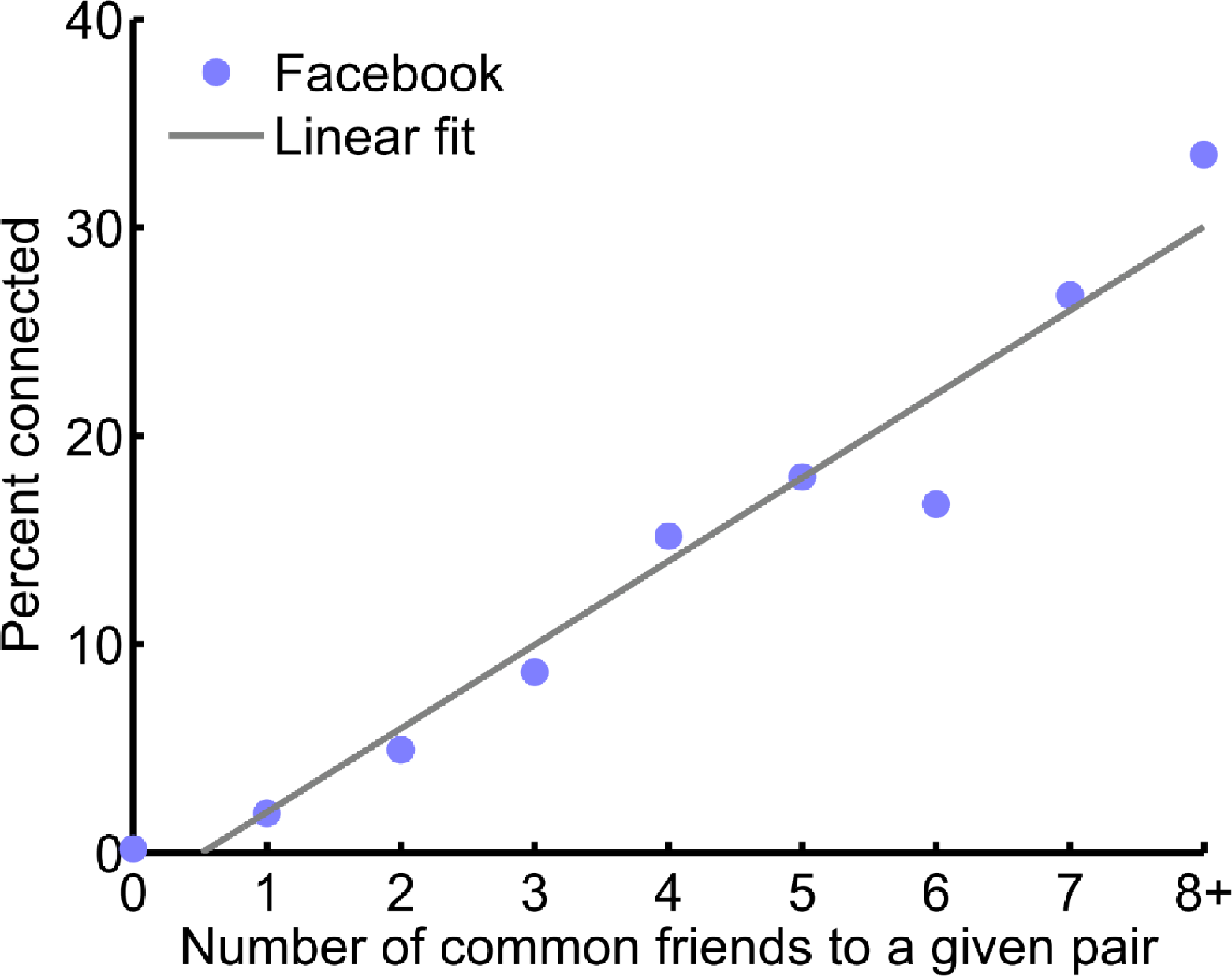

Supplement: S9 Fig — The data is based on the Stanford Large Network Dataset Collection and can be downloaded from: http://snap.stanford.edu/data/egonets-Facebook.html. This network is an anonymized Facebook ego-network of a single user with all his/her friends together with all the connections among these friends. We compiled a network of 348 users that we analyzed in the same way as we analyzed the C. elegans neural network (Fig 1B). We found that the CNR is a significant property of social networks as well (linear fit, R2 = 0.95). (TIF) [file pcbi.1005021.s010.tif]
