# Supplementary material for: The C. elegans Connectome Consists of Homogenous Circuits with Defined Functional Roles
Source: PLoS Comput Biol. 2016 Sep 8;12(9):e1005021. doi: 10.1371/journal.pcbi.1005021 (PMC5015834; doi:10.1371/journal.pcbi.1005021)
Supplement: S1 Table — Data compiled based on: http://www.wormatlas.org/neurons/Individual%20Neurons/Neuronframeset.html (PDF) [file pcbi.1005021.s011.pdf]

Supplementary Table S1. A list of all neurons together with their assigned layer in the network (Sensory, Inter, Pre-Motor, Motor). Data compiled based on: http://www.wormatlas.org/neurons/Individual%20Neurons/Neuronframeset.html

Neuron	Level	Neuron	Level	Neuron	Level	Neuron	Level	Neuron	Level	Neuron	Level
ADAL	Inter	ASJL	Sensory	DA09	Motor	PHBL	Sensory	RMDR	Motor	URYDR	Sensory
ADAR	Inter	ASJR	Sensory	DB01	Motor	PHBR	Sensory	RMDVL	Motor	URYVL	Sensory
ADEL	Sensory	ASKL	Sensory	DB02	Motor	PHCL	Sensory	RMDVR	Motor	URYVR	Sensory
ADER	Sensory	ASKR	Sensory	DB03	Motor	PHCR	Sensory	RMED	Motor	VA01	Motor
ADFL	Sensory	AUAL	Inter	DB04	Motor	PLML	Sensory	RMEL	Motor	VA02	Motor
ADFR	Sensory	AUAR	Inter	DB05	Motor	PLMR	Sensory	RMER	Motor	VA03	Motor
ADLL	Sensory	AVAL	Pre-motor	DB06	Motor	PLNL	Inter	RMEV	Motor	VA04	Motor
ADLR	Sensory	AVAR	Pre-Motor	DB07	Motor	PLNR	Inter	RMFL	Motor	VA05	Motor
AFDL	Sensory	AVBL	Pre-Motor	DD01	Motor	PQR	Sensory	RMFR	Motor	VA06	Motor
AFDR	Sensory	AVBR	Pre-Motor	DD02	Motor	PVCL	Pre-Motor	RMGL	Inter	VA07	Motor
AIAL	Inter	AVDL	Pre-Motor	DD03	Motor	PVCR	Pre-Motor	RMGR	Inter	VA08	Motor
AIAR	Inter	AVDR	Pre-Motor	DD04	Motor	PVDL	Sensory	RMHL	Motor	VA09	Motor
AIBL	Inter	AVEL	Pre-Motor	DD05	Motor	PVDR	Sensory	RMHR	Motor	VA10	Motor
AIBR	Inter	AVER	Pre-Motor	DD06	Motor	PVM	Sensory	SAADL	Motor	VA11	Motor
AIML	Inter	AVFL	Inter	DVA	Inter	PVNL	Motor	SAADR	Motor	VA12	Motor
AIMR	Inter	AVFR	Inter	DVB	Motor	PVNR	Motor	SAAVL	Motor	VB01	Motor
AINL	Inter	AVG	Inter	DVC	Inter	PVPL	Inter	SAAVR	Motor	VB02	Motor
AINR	Inter	AVHL	Inter	FLPL	Sensory	PVPR	Inter	SABD	Motor	VB03	Motor
AIYL	Inter	AVHR	Inter	FLPR	Sensory	PVQL	Inter	SABVL	Motor	VB04	Motor
AIYR	Inter	AVJL	Inter	HSNL	Motor	PVQR	Inter	SABVR	Motor	VB05	Motor
AIZL	Inter	AVJR	Inter	HSNR	Motor	PVR	Inter	SDQL	Inter	VB06	Motor
AIZR	Inter	AVKL	Inter	IL1DL	Sensory	PVT	Inter	SDQR	Inter	VB07	Motor
ALA	Inter	AVKR	Inter	IL1DR	Sensory	PVWL	Inter	SIADL	Motor	VB08	Motor
ALML	Sensory	AVL	Motor	IL1L	Sensory	PVWR	Inter	SIADR	Motor	VB09	Motor
ALMR	Sensory	AVM	Sensory	IL1R	Sensory	RIAL	Inter	SIAVL	Motor	VB10	Motor
ALNL	Sensory	AWAL	Sensory	IL1VL	Sensory	RIAR	Inter	SIAVR	Motor	VB11	Motor
ALNR	Sensory	AWAR	Sensory	IL1VR	Sensory	RIBL	Inter	SIBDL	Motor	VC01	Motor
AQR	Sensory	AWBL	Sensory	IL2DL	Sensory	RIBR	Inter	SIBDR	Motor	VC02	Motor
AS01	Motor	AWBR	Sensory	IL2DR	Sensory	RICL	Inter	SIBVL	Motor	VC03	Motor
AS02	Motor	AWCL	Sensory	IL2L	Sensory	RICR	Inter	SIBVR	Motor	VC04	Motor
AS03	Motor	AWCR	Sensory	IL2R	Sensory	RID	Inter	SMBDL	Motor	VC05	Motor
AS04	Motor	BAGL	Sensory	IL2VL	Sensory	RIFL	Inter	SMBDR	Motor	VD01	Motor
AS05	Motor	BAGR	Sensory	IL2VR	Sensory	RIFR	Inter	SMBVL	Motor	VD02	Motor
AS06	Motor	BDUL	Inter	LUAL	Inter	RIGL	Inter	SMBVR	Motor	VD03	Motor
AS07	Motor	BDUR	Inter	LUAR	Inter	RIGR	Inter	SMDDL	Motor	VD04	Motor
AS08	Motor	CEPDL	Sensory	OLLL	Sensory	RIH	Inter	SMDDR	Motor	VD05	Motor
AS09	Motor	CEPDR	Sensory	OLLR	Sensory	RIML	Motor	SMDVL	Motor	VD06	Motor
AS10	Motor	CEPVL	Sensory	OLQDL	Sensory	RIMR	Motor	SMDVR	Motor	VD07	Motor
AS11	Motor	CEPVR	Sensory	OLQDR	Sensory	RIPL	Inter	URADL	Motor	VD08	Motor
ASEL	Sensory	DA01	Motor	OLQVL	Sensory	RIPR	Inter	URADR	Motor	VD09	Motor
ASER	Sensory	DA02	Motor	OLQVR	Sensory	RIR	Inter	URAVL	Motor	VD10	Motor
ASGL	Sensory	DA03	Motor	PDA	Motor	RIS	Inter	URAVR	Motor	VD11	Motor
ASGR	Sensory	DA04	Motor	PDB	Motor	RIVL	Inter	URBL	Inter	VD12	Motor
ASHL	Sensory	DA05	Motor	PDEL	Sensory	RIVR	Inter	URBR	Inter	VD13	Motor
ASHR	Sensory	DA06	Motor	PDER	Sensory	RMDDL	Motor	URXL	Inter		
ASIL	Sensory	DA07	Motor	PHAL	Sensory	RMDDR	Motor	URXR	Inter		
ASIR	Sensory	DA08	Motor	PHAR	Sensory	RMDL	Motor	URYDL	Sensory		
