# Supplementary material for: The C. elegans Connectome Consists of Homogenous Circuits with Defined Functional Roles
Source: PLoS Comput Biol. 2016 Sep 8;12(9):e1005021. doi: 10.1371/journal.pcbi.1005021 (PMC5015834; doi:10.1371/journal.pcbi.1005021)
Supplement: S2 Table — The first row is the neuron name, and the second is the triad type of a given Z neuron. (PDF) [file pcbi.1005021.s012.pdf]

Supplementary Table S2. A list of the most significant homogeneous common neighbor sets (Hypergeometric test p-value threshold = 10^{-5}). The first row is the neuron names, and the second is the triad type of a given Z neuron.

X	Y	Z1	Z2	Z3	Z4	Z5	Z6	Z7	Z8	Z9	Z10	Z11	Z12	Z13	Z14	Z15	Z16	Z17	Z18	Z19	Z20	Z21	Z22	Z23
ADAL	FLPR	AIBR	AVAR	AVBL	AVBR	AVDL	AVDR	AVEL	AVJR															
		1	1	1	1	3	9	1	1															
ADLL	ADLR	AIBR	ASER	AVAR	AVBL	AVDL	AVDR	AVHR	AVJR															
		10	10	10	10	10	12	13	10															
ADLR	ASHR	AIAR	AIBR	AVAR	AVBR	AVDL	AVDR	RID																
		8	1	1	1	1	1	3																
AVHR	ADLR	ADLL	AVBL	AVBR	AVDR	AVJR																		
		3	1	1	1	1																		
AIAR	AIBR	ADAR	ADLR	AIZR	ASER	ASGR	ASHR	ASIR	AWCL	AWCR														
		2	7	7	7	7	5	7	7	7														
AIBR	AVAR	ADAL	ADLL	ADLR	ASHR	AVBR	AVEL	DVC	FLPL	FLPR	RIGR	RIMR	RIPL	SAAVL	SDQR									
		5	5	5	5	2	3	7	5	5	8	9	3	5	5									
AIML	AVBR	AVFL	AVFR	AVHL	AVHR	AVJL	RIFR																	
		2	2	2	2	2	2																	
AIZR	DVA	AIZL	AVEL	AVER	RIBL	RIPL	RIS	SMBVL	SMDDL															
		9	1	1	1	1	6	1	1															
VD06	AVAL	AS06	DA05	DA06	PVPL	VA07																		
		6	6	6	5	9																		
PHBR	AVAR	AVAL	AVDL	AVDR	DA08	PHBR	PVCR	PVDL	VB01															
		3	3	3	3	7	3	3	3															
VD05	AVAR	AS05	DA04	DA05	DA06	DB03	PVQL	VA06	VA07															
		6	6	6	6	4	7	9	6															
AVJR	AVBL	ADAL	ADLL	ADLR	AVAL	AVAR	AVBR	AVDL	AVDR	AVER	AVFL	AVFR	AVHR	AVJL	AVM	FLPR	PVCR	PVDL	PVPL	PVR	PVT	RIFR	RIGL	URXR
		5	5	5	1	3	3	1	3	1	5	5	5	7	5	5	7	2	6	2	5	5	5	5
RIFR	AVBL	AVBR	AVFL	AVG	AVHL	AVJL	AVJR	PVCR	PVDL	PVQL	PVR	RIPL												
		3	2	7	2	7	2	2	2	2	5	2												
AVJR	AVBR	ADAL	ADLR	AVAL	AVAR	AVBL	AVFL	AVFR	AVHR	AVJL	AVM	FLPR	PVCR	PVDL	PVNR	PVPL	PVT	RIFR	RIGL					
		5	5	3	1	3	5	5	5	7	5	5	7	2	6	5	5	5	5					
RIFR	AVBR	ASHR	AVBL	AVFL	AVG	AVHL	AVJL	AVJR	HSNR	PVCR	PVDL	PVQL	RIPL											
		2	3	2	7	2	7	2	8	2	2	2	2											
AVDL	AVM	AVBL	AVJR	DA01	PVCR	PVDL	PVNR																	
		11	11	10	12	11	11																	
AVG	PVPR	AVAR	AVBL	AVBR	AVL	PVCR	PVR	RIGL																
		1	1	1	3	1	1	7																
RIS	AVKR	AVKL	RIMR	RIPL	RMDVL	SMDDR	SMDVL																	
		3	8	8	8	8	8																	
CEPDR	URYDR	AVEL	RICL	RMDDR	RMED	URAVL																		
		1	1	1	1	2																		
DD01	DD02	RIFL	VA04	VB03	VC02	VC03	VC04																	
		13	13	13	13	13	13																	
DD01	VD02	AS02	AS03	DA01	DA02	DB01	VA03	VA04	VB02	VB03	VC02	VC03	VC04	VD02										
		13	13	13	14	13	14	13	13	14	13	13	13	15										
DD02	DD03	DB03	RIFL	VA06	VB05	VC02	VC03	VC04																
		13	13	13	13	13	13	13																
IL2DR	URADR	CEPDR	IL1DR	RIR	RMEL	RMEV																		
		2	1	1	1	1																		
IL2VL	RIH	BAGR	IL2L	OLQVR	RIAR	RIPR	RMEV	RMFL																
		1	3	9	1	1	1	1																
LUAL	PVNL	AVAL	AVDL	AVDR	AVJL	PVWR																		
		8	8	8	1	1																		
PHAL	PHAR	AVG	AVHR	DVA	PHBR	PHCL	PVQR																	
		12	10	10	10	10	10																	
RMEL	RMEV	IL2DR	IL2R	IL2VL	IL2VR	URAVR																		
		13	13	13	13	13																		
RMER	RMEV	IL2DR	IL2L	IL2VL	IL2VR	RIML	URAVR	URBL																
		13	13	13	13	13	13	13																
VA08	VA09	AVAL	AVAR	AVBR	DD04	PVDL	VB09	VB10	VD08															
		9	7	9	1	9	9	9	3															
VC01	VC02	DD01	DD02	DD03	DVC	PVWL	VC04	VD02	VD03	VD05	VD06	VD07												
		10	10	10	10	10	15	12	10	10	10	10												
VC01	VC03	AVL	DD01	DD02	DD03	DVC	VC03	VC05	VD02	VD03	VD04	VD05	VD06	VD07										
		10	10	10	10	10	15	14	12	10	10	10	10	10										
VC02	VC03	DD01	DD02	DD03	DVC	HSNR	PVPL	PVR	VC02	VD02	VD03	VD05	VD06	VD07										
		10	10	10	10	14	14	10	15	10	10	10	10	10										
VD04	VD05	DA04	DA06	DB02	DB03	PVQL	VC02	VC03	VC04															
		13	13	13	13	15	13	13	13															
VD05	VD06	DA05	DA06	DB03	VA07	VC02	VC03	VC04																
		13	13	13	14	13	13	13																
